# ABHD11-AS1 Suppresses Colorectal Cancer Progression by Disrupting EIF4E-mediated POU2F1 Ubiquitination

**DOI:** 10.7150/ijbs.125799

**Published:** 2026-04-23

**Authors:** Shizhen Li, Xianjie Jiang, Linda Oyang, Longzheng Xia, Shiming Tan, Zongyao Ren, Qiu Peng, Jinguan Lin, Qianjin Liao, Yujuan Zhou

**Affiliations:** 1 Hunan Key Laboratory of Cancer Metabolism, Hunan Cancer Hospital and the Affiliated Cancer Hospital of Xiangya School of Medicine, Central South University, Changsha, 410013, Hunan, China.; 2 Hunan Engineering Research Center of Tumor organoid Technology and application, Public Service Platform of Tumor organoids Technology, 283 Tongzipo Road, Changsha, 410013, Hunan, China.; 3 Department of Oncology, Hunan Provincial People's Hospital and the First Affiliated Hospital of Hunan normal university, Hunan Normal University Health Science Center, Changsha, 410005, Hunan, China.

**Keywords:** colorectal cancer, ABHD11-AS1, EIF4E, phase separation, POU2F1, ubiquitination

## Abstract

Colorectal cancer (CRC) remains one of the leading causes of cancer-related mortality worldwide, yet the underlying mechanisms driving its progression are not fully elucidated. Long non-coding RNAs (lncRNAs) have recently emerged as key regulatory molecules in tumor biology. In this study, we identified ABHD11-AS1 as a tumor-suppressive lncRNA that is significantly downregulated in CRC tissues, its low expression is correlated with poor patient prognosis. Functional assays demonstrated that ABHD11-AS1 inhibits CRC cell proliferation, migration, and invasion, and enhances sensitivity to oxaliplatin. Mechanistically, ABHD11-AS1 directly binds to EIF4E and disrupts its phase separation, thereby suppressing the translation of USP18, a deubiquitinating enzyme that stabilises the oncogenic protein POU2F1. Reduced USP18 expression leads to increased ubiquitination and proteasomal degradation of POU2F1, ultimately inhibiting malignant progression and enhancing chemotherapy sensitivity. Collectively, our findings uncover a previously unrecognised mechanism by which ABHD11-AS1 modulates EIF4E-mediated phase separation to regulate protein homeostasis, highlighting its potential as a therapeutic target in CRC.

## Introduction

Colorectal cancer (CRC), a malignancy of the lower gastrointestinal tract, is marked by high invasiveness and metastatic potential. It ranks as the third most commonly diagnosed cancer worldwide, with both incidence and mortality steadily increasing in recent years—particularly among younger individuals—posing a growing global public health challenge 1-5. Despite significant advances in surgical resection, chemotherapy, targeted therapy, and immunotherapy, the overall prognosis for patients with CRC remains poor—particularly for those with advanced-stage disease, whose five-year survival rate falls below 20% 6, 7. Elucidating the molecular mechanisms driving CRC progression and treatment resistance is therefore critical for identifying novel therapeutic targets and improving clinical outcomes.

Long non-coding RNAs (lncRNAs), a class of transcripts over 200 nucleotides in length with limited protein-coding capacity, have recently emerged as critical regulators of cancer biology. They regulate protein-coding gene expression at multiple levels, including transcriptional, translational, and post-translational processes 8-13. Extensive studies have shown that lncRNAs can function as either oncogenes or tumor suppressors through diverse mechanisms, including acting as molecular sponges for microRNAs to derepress downstream targets, or directly interacting with proteins to regulate their activity and stability 14-21. Loss of tumor-suppressive lncRNAs is frequently associated with enhanced malignancy and treatment resistance. For example, depletion of LINC01056, CRNDE, and LINC01852 has been linked to disease progression and chemoresistance in several cancer types, including CRC 22-29. Nevertheless, the roles and underlying mechanisms of lncRNAs in CRC remain incompletely understood.

POU class 2 homeobox 1 (POU2F1, also known as OCT1) is a key transcription factor that regulates oxidative stress responses, metabolic reprogramming, and stemness maintenance in tumor cells 30-32. Our previous studies have shown that POU2F1 is highly expressed in CRC, where it promotes tumor progression and confers oxaliplatin resistance by facilitating glucose metabolic reprogramming in cancer cells 33. However, the regulatory mechanisms controlling POU2F1 expression and activity in CRC remain largely unclear. Although several studies have shown that specific lncRNAs can modulate POU2F1 expression and function in other cancer types, systematic investigations into their roles in CRC are still lacking 34-36.

Phase separation refers to the biophysical process by which biomolecules, such as proteins and RNAs, undergo multivalent interactions to form distinct, membrane-less compartments with liquid-like properties. This phase separation creates concentrated molecular assemblies-often termed condensates-that enable spatial and temporal regulation of cellular processes, including transcription, RNA metabolism, signal transduction, and stress responses 37-39. Recent studies have shown that phase separation also plays an important role in regulating mRNA translation. Primarily by facilitating the dynamic assembly of translation initiation factors, ribosomes and mRNAs into membrane-free condensates, phase separation enhances the efficiency and fine-tuned control of protein synthesis, especially under tumor-related stress conditions 40-42. However, the upstream regulatory mechanisms governing phase separation remain poorly understood and warrant further investigation.

In this study, we identified the ABHD11-AS1 as a tumor-suppressive lncRNA in CRC, characterized by significantly reduced expression in tumor tissues and a strong association with poor patient prognosis. ABHD11-AS1 suppresses CRC growth, metastasis and enhances the sensitivity of cancer cells to oxaliplatin via downregulating POU2F1. Mechanistically, ABHD11-AS1 binds to and disrupts EIF4E phase separation, thereby reducing the translational efficiency of the deubiquitinating enzyme USP18. This leads to diminished deubiquitination of its target, POU2F1, promoting its ubiquitin-mediated degradation and ultimately suppressing CRC progression while enhancing oxaliplatin sensitivity. Our findings provide new insights into the role of lncRNA-mediated phase separation in translational regulation and offer a novel therapeutic target and strategy for the precision treatment of CRC.

## Materials and Methods

### Human tissue samples

All participants provided written informed consent prior to enrollment. This study was approved by the Ethics Committee of Hunan Cancer Hospital. Between 2007 and 2011, a total of 78 surgical specimens were collected from patients diagnosed with CRC at Hunan Cancer Hospital. Additionally, 39 matched pairs of adjacent normal tissue samples were obtained. All specimens were fixed in formalin and embedded in paraffin for subsequent analyses.

### Cell lines and cell culture

The human normal colon epithelial cell line NCM460 (cat. no. CL0393) and six colon cancer cell lines, HCT116 (cat. no. CL0125), HT29 (cat. no. CL0163), SW480 (cat. no. CL0303), SW620 (cat. no. CL0305), HCT8 (cat. no. CL0127), and LOVO (cat. no. CL0197), along with HEK293 cells (cat. no. CL0132), were obtained from Hunan Fenghui Biotechnology. All cells were identified by STR. Detailed cell culture conditions were as previously described 43. Briefly, NCM460, HCT116 and HEK293 cells were cultured in Dulbecco's Modified Eagle Medium (DMEM; Gibco, USA) supplemented with 10% fetal bovine serum (FBS; ZETA LIFE, USA). HT29, SW480, SW620, HCT8, and LOVO cells were cultured in RPMI-1640 medium (Gibco, USA) supplemented with 10% FBS.

### Plasmids, siRNA, and cell transfection

Overexpression plasmids for ABHD11-AS1 and USP18, along with three short hairpin RNAs (shRNAs) targeting ABHD11-AS1 (sh-ABHD11-AS1-1, sh-ABHD11-AS1-2, sh-ABHD11-AS1-3) and USP18 (sh-USP18-1, sh-USP18-2, sh-USP18-3), were purchased from GeneChem (Shanghai, China). The corresponding sequences are listed in Supplementary Table S1. The overexpression plasmid and shRNA targeting POU2F1 were the same as those used in our previous study 33. Transfection of HCT116, SW620, and HEK293 cells was performed using Lipofectamine™ 3000 (Invitrogen, USA), according to the manufacturer's protocol. The efficiency of gene overexpression and knockdown was assessed by qRT-PCR.

### Cell viability and colony formation assays

Cell viability and colony formation assays were conducted as previously described 33. Briefly, proliferating cells (5,000 cells per well) were seeded into 96-well plates, and cell viability was measured using the Cell Counting Kit-8 (CCK-8; ZETA LIFE, K009-500). Each group included five technical replicates. For the colony formation assay, 2,000 transfected cells per dish were seeded into 6 cm culture dishes and incubated for two weeks. Colonies were then fixed and stained with 0.1% crystal violet and manually counted. The evaluation of colony numbers was performed in a blinded manner.

### Transwell migration, invasion, and wound healing assays

Transwell chambers (8.0μm pore size; 3422, Costar) were used for both migration and invasion assays. For the migration assay, 200μL of RPMI-1640 medium containing 2×10⁴ transfected cells was added to the upper chamber, while 600 μL of RPMI-1640 medium supplemented with 20% fetal bovine serum (FBS) was placed in the lower chamber. For the invasion assay, the upper chamber was first pre-coated with 50μL of Matrigel (356230, BD Pharmingen) per well. Then, 200μL of RPMI-1640 medium containing 2×10⁴ cells was added to the upper chamber, and 600μL of RPMI-1640 medium with 20% FBS was added to the lower chamber. After incubation for 48 hours, cells that had invaded through the membrane were fixed with paraformaldehyde, non-invading cells in the upper chamber were removed, and the remaining cells were stained and counted under a light microscope. For the wound healing assay, cells were seeded in 6-well plates and grown to confluence. A scratch was created using a sterile 10μL pipette tip. After washing with PBS to remove detached cells, the monolayer was cultured in serum-free medium for 48 hours. Images were captured at 0 and 48 hours using a Zeiss light microscope.

### Annexin V-PE / 7-ADD staining for apoptosis detection

Cell apoptosis was assessed using the Annexin V-PE/7-ADD apoptosis detection kit (MA0429, Merlunbio). After transfection, cells were treated with 5μM oxaliplatin and subsequently harvested. Following two washes with PBS, cells were resuspended in 500μL of binding buffer, and 5μL of Annexin V-PE and 5μL of 7-ADD dye were added. Samples were analyzed using a CytoFLEX flow cytometer. Cells negative for both PE and 7-ADD (PE⁻/7-ADD⁻) were considered viable, cells positive for PE but negative for 7-ADD (PE⁺/7-ADD⁻) were classified as early apoptotic, and cells positive for both PE and 7-ADD (PE⁺/7-ADD⁺) were identified as late apoptotic or necrotic.

### RNA extraction and qRT-PCR

Total RNA was extracted using TRIzol reagent (15596-018; Invitrogen) as previously described 33. Complementary DNA (cDNA) synthesis was performed using the iScript™ cDNA Synthesis Kit (1708891, Bio-Rad). qRT-PCR was conducted on a Roche LightCycler® 96 system (Lifescience) using the SYBR Green Pro Taq HS qPCR Kit (AG11701, Accurate Biology). Primer sequences are listed in Supplementary Table S2. The PCR protocol included an initial denaturation at 95°C for 30 seconds, followed by 40 amplification cycles (95°C for 5 seconds and 60°C for 30 seconds). Gene expression levels were calculated using the 2^-ΔΔCt^ method. U6 was used as the internal control for nuclear transcripts, while ACTB served as the reference gene for cytoplasmic transcripts.

### Western blot analysis

Western blotting was performed to assess the relative expression levels of target proteins. A total of 30μg of protein from cell lysates was separated via 10% SDS-PAGE and transferred onto polyvinylidene fluoride membranes. Membranes were blocked with QuickBlock™ Blocking Buffer (P0252, Beyotime) and incubated with primary antibodies overnight at 4°C. After incubation with appropriate secondary antibodies, signals were detected using the Pierce™ ECL Western Blotting Substrate (Thermo Scientific). Protein bands were quantified using ImageJ software. Detailed information on the antibodies used is provided in Supplementary Table S3.

### Immunocytochemistry

Adherent cells were fixed with 4% paraformaldehyde for 15 minutes and permeabilized with 0.5% Triton X-100 for 10 minutes. After blocking with 1% BSA in PBS at room temperature for 1 hour, cells were incubated overnight at 4°C with primary antibodies (listed in Supplementary Table S3). The following day, cells were washed and incubated with secondary antibodies at room temperature for 1 hour. Nuclei were counterstained with DAPI (Sigma) for 5 minutes. After mounting with antifade medium, images were captured using a confocal laser scanning microscope (LSM880 with Fast Airyscan, ZEISS).

### RNA immunoprecipitation

RNA immunoprecipitation (RIP) assays were performed using the Magna RIP Kit (17-701, Millipore) following the manufacturer's instructions. Briefly, HCT116 and SW620 cells were lysed and centrifuged at 14,000 rpm for 10 minutes at 4°C. A 10μL aliquot of the supernatant was reserved as input, and the remaining supernatant was incubated overnight at 4°C with the primary antibody and pre-washed Protein A/G magnetic beads. After washing the immunoprecipitates, samples were treated with Proteinase K buffer at 55°C for 30 minutes. The RNA was then purified and analyzed by RT-PCR.

### In situ hybridization

In situ hybridization (ISH) was performed as previously described. A specific probe targeting ABHD11-AS1 (listed in Supplementary Table S2; Sangon Biotech, Shanghai, China) was used to detect its expression in CRC tissue specimens. The staining results were evaluated based on both staining intensity and the proportion of positively stained cells, following the scoring criteria described in reference.

### Nuclear and cytoplasmic fractionation

Nuclear and cytoplasmic fractions of HCT116 and SW620 cells were separated using the PARIS™ Kit (AM1921, Invitrogen) according to the manufacturer's instructions. Following RNA extraction from each compartment, quantitative real-time PCR (qRT-PCR) was performed to assess RNA distribution. U6 was used as the nuclear control, while GAPDH served as the cytoplasmic control.

### RNA pull down assay

The RNA pull-down assay was performed using the RNAmax-T7 Biotinylated Transcription Kit (RiboBio Biotechnology, China). A linear DNA template containing the T7 promoter was designed and amplified by PCR. *In vitro* transcription was then carried out to synthesize biotin-labeled RNA, followed by purification. RNA-binding proteins were captured using M-280 streptavidin magnetic beads (11205D, Invitrogen, USA), and the associated target proteins were subsequently detected by Western blot analysis. Primer sequences used for transcription are listed in Supplementary Table S2.

### Fluorescence recovery after photobleaching

The GFP-tagged EIF4E plasmid was purchased from GeneChem (Shanghai, China). To evaluate the dynamic properties of EIF4E condensates and assess the impact of ABHD11-AS1 on its phase separation behavior, a fluorescence recovery after photobleaching (FRAP) assay was conducted. Briefly, the GFP-tagged EIF4E construct was transfected into HEK293T cells, which were then seeded onto 35 mm glass-bottom dishes (Cellvis). Forty-eight hours post-transfection, live-cell imaging was performed using a confocal laser scanning microscope (e.g., Zeiss LSM880) with a 63× oil immersion objective. Regions of interest (ROIs) within the GFP-labeled condensates were selected and photobleached using a high-intensity 488 nm laser for 1-2 seconds. Fluorescence recovery within the bleached area was subsequently recorded at 1-2 second intervals by continuous image acquisition.

### Statistical analysis

Statistical analyses were performed using GraphPad Prism (version 9.5) and SPSS (version 19.0; IBM Corp.). Comparisons between groups were conducted using two-tailed Student's *t*-test, log-rank test, Spearman's correlation analysis, and one-way or two-way analysis of variance (ANOVA), as appropriate. Data are presented as mean ± standard deviation (SD). Statistical significance was defined as follows: *P* ≤ 0.05 (*), *P* < 0.01 (**), *P* < 0.001 (***), and *P* < 0.0001 (****).

## Results

### ABHD11-AS1 is downregulated in colorectal cancer and is associated with poor patient prognosis

**Figure 1:**
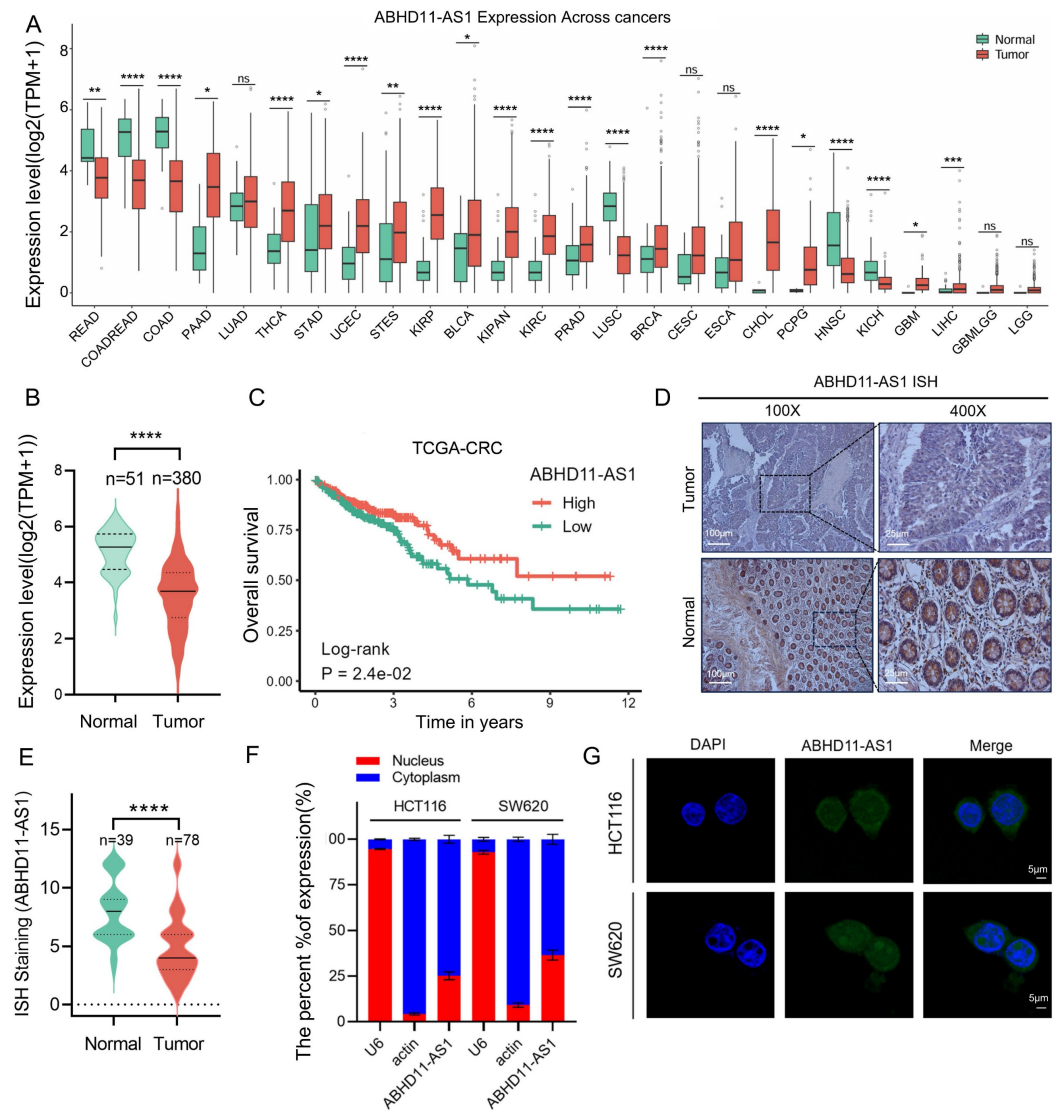
** ABHD11-AS1 is downregulated in colorectal cancer and correlates with poor patient prognosis.** (A) Pan-cancer expression analysis of ABHD11-AS1 based on the TCGA Pan-Cancer dataset obtained from UCSC Xena. Expression data for ABHD11-AS1 were extracted, log2(x+1) transformed, and compared between tumor and adjacent normal tissues. Only cancer types with ≥3 samples were included. (B) The expression levels of ABHD11-AS1 were analyzed by comparing tumor and normal tissues using TCGA data. (C) Kaplan-Meier survival analysis of OS in CRC patients stratified by ABHD11-AS1 expression levels, based on TCGA datasets. (D, E) ISH was used to assess the expression levels of ABHD11-AS1 in CRC tissues and adjacent normal tissues from clinical samples. (F, G) Subcellular localization of ABHD11-AS1 was examined using FISH combined with nucleocytoplasmic fractionation assays. Magnification, ×100, scale bar = 100 μm; Magnification, ×400, scale bar = 20 μm; Confocal microscopy, scale bar = 5μm; ns, not significant; *P < 0.05; **P < 0.01; ***P < 0.001; ****P < 0.0001.

ABHD11-AS1 (also known as ABHD11 antisense RNA 1) is a long non-coding RNA consisting of 473 bases and located on human chromosome 7q11.23 (Supplementary Figure S1A) 44. Previous studies have demonstrated that ABHD11-AS1 exerts important biological functions in various cancers; however, its role in CRC remains unclear 45, 46. To investigate the expression profile of ABHD11-AS1 across cancer types, we analyzed normalized pan-cancer RNA-sequencing data (10,535 samples, 60,499 genes) from The Cancer Genome Atlas (TCGA) downloaded via the UCSC Xena platform (https://xenabrowser.net/). Expression data for ABHD11-AS1 (ENSG00000225969) were extracted and log_2_(x+1)-transformed for analysis. Only cancer types with ≥3 samples were included, yielding a final dataset encompassing 26 cancer types. The results revealed that ABHD11-AS1 was significantly downregulated in colon adenocarcinoma (COAD) and rectal adenocarcinoma (READ) compared to normal tissues (Figure 1A-B; Supplementary Figure S1B). Kaplan-Meier survival analysis based on TCGA datasets showed that low ABHD11-AS1 expression was significantly associated with shorter overall survival in CRC patients (Figure 1C). To investigate the expression of ABHD11-AS1 in clinical CRC tissues, ISH was performed on 78 clinical CRC tissue samples and 39 normal colorectal mucosa samples. The results showed that ABHD11-AS1 expression was markedly downregulated in CRC tissues (Figure 1D-E). In addition, the subcellular localization of ABHD11-AS1 were examined using nuclear-cytoplasmic fractionation and fluorescence FISH assays. Results showed that ABHD11-AS1 was distributed in both the nucleus and cytoplasm (Figure 1F-G). In summary, our findings demonstrate that ABHD11-AS1 is significantly downregulated in CRC and is associated with poor patient prognosis, suggesting its potential role in colorectal tumorigenesis and its promise as a prognostic biomarker for CRC.

### ABHD11-AS1 inhibits colorectal cancer progression and promotes oxaliplatin sensitivity

**Figure 2:**
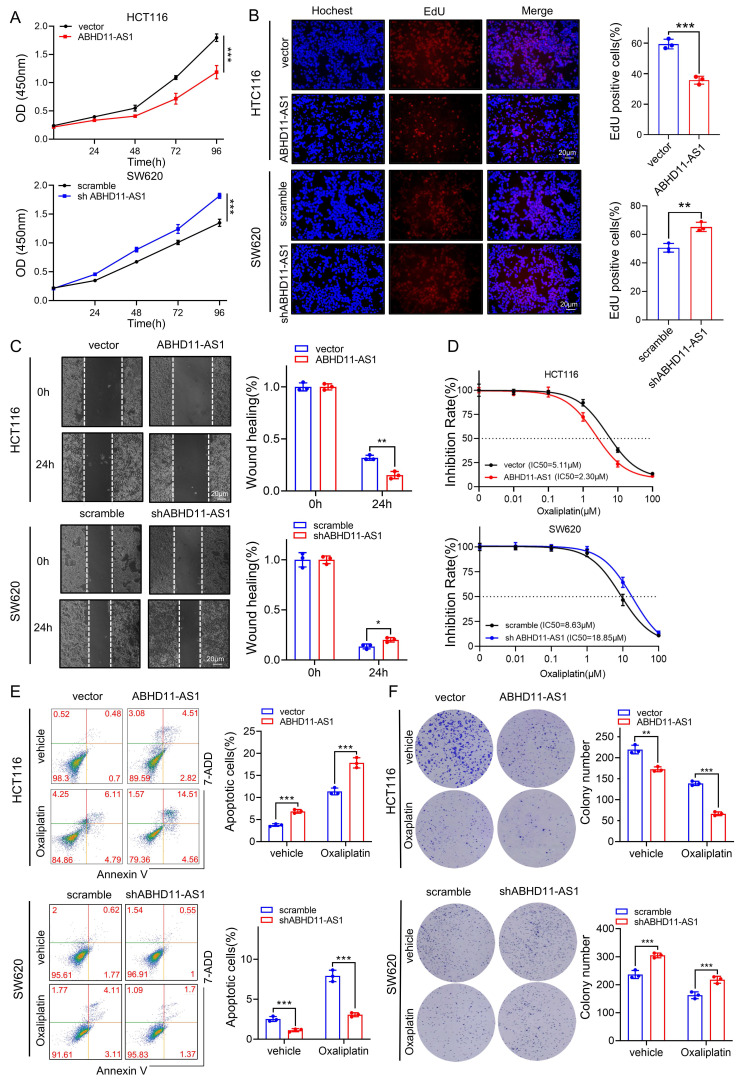
** ABHD11-AS1 suppresses CRC proliferation and migration and enhances sensitivity to oxaliplatin.** (A, B) CRC cell proliferation was evaluated using the CCK-8 assay and EdU incorporation assay. (C) CRC cell migration was evaluated using wound healing assay. (D) The effect of ABHD11-AS1 on CRC cell sensitivity to oxaliplatin was assessed by treating cells with gradient concentrations of oxaliplatin followed by IC50 determination. (E, F) The effect of ABHD11-AS1 on CRC cell sensitivity to oxaliplatin was evaluated by flow cytometry-based apoptosis assay and colony formation assay following treatment with or without 5μM oxaliplatin. Data are presented as the mean ± SD from three independent experiments. Magnification, ×200; scale bar = 20μm; *P < 0.05; **P < 0.01; ***P < 0.001.

To further investigate the biological role of ABHD11-AS1 in CRC cells, we constructed three shRNAs targeting ABHD11-AS1 and an ABHD11-AS1 overexpression plasmid. qRT-PCR analysis were performed to examine the expression of ABHD11-AS1 in overexpression or knock down experiments (Supplementary Figure S2A). Of the three shRNAs tested, shABHD11-AS1-3 exhibited the highest knockdown efficiency and was thus selected for all subsequent experiments. CCK-8, EdU and colony formation assays showed that ABHD11-AS1 overexpression significantly inhibited the proliferation in HCT116 cells, whereas knockdown of ABHD11-AS1 markedly promoted the proliferation in SW480 cells (Figure 2A-B; Supplementary Figure S2B). Wound healing assays further revealed that ABHD11-AS1 overexpression impaired the migratory ability of HCT116 cells, while its knockdown promoted SW620 cell migration (Figure 2C). Consistently, Transwell migration and invasion assays validated the regulatory role of ABHD11-AS1 in cell motility and invasiveness (Supplementary Figure S2C). Chemoresistance is a major driver of tumor relapse and poor prognosis. Loss of specific lncRNAs has been implicated in promoting resistance, underscoring the urgent need to overcome this barrier in clinical therapy 27-29. Oxaliplatin, a platinum-based compound, remains a cornerstone of first-line therapy for CRC, yet resistance frequently limits its clinical efficacy. Emerging evidence indicates that lncRNAs modulate oxaliplatin sensitivity through diverse molecular mechanisms 47-49. To evaluate the role of ABHD11-AS1 in oxaliplatin resistance, we performed flow cytometry, IC_50_ measurements, and colony formation assays following oxaliplatin treatment. The results showed ABHD11-AS1 overexpression enhanced oxaliplatin responsiveness in HCT116 cells, whereas its knockdown conferred resistance in SW620 cells (Figure 2D-F). Collectively, these findings demonstrate that ABHD11-AS1 suppresses CRC cell proliferation, migration and invasion, while enhancing sensitivity to oxaliplatin. These results highlight ABHD11-AS1 as a potential therapeutic target with clinical relevance in CRC.

### POU2F1 functions as a key downstream effector in ABHD11-AS1-mediated colorectal cancer progression

**Figure 3:**
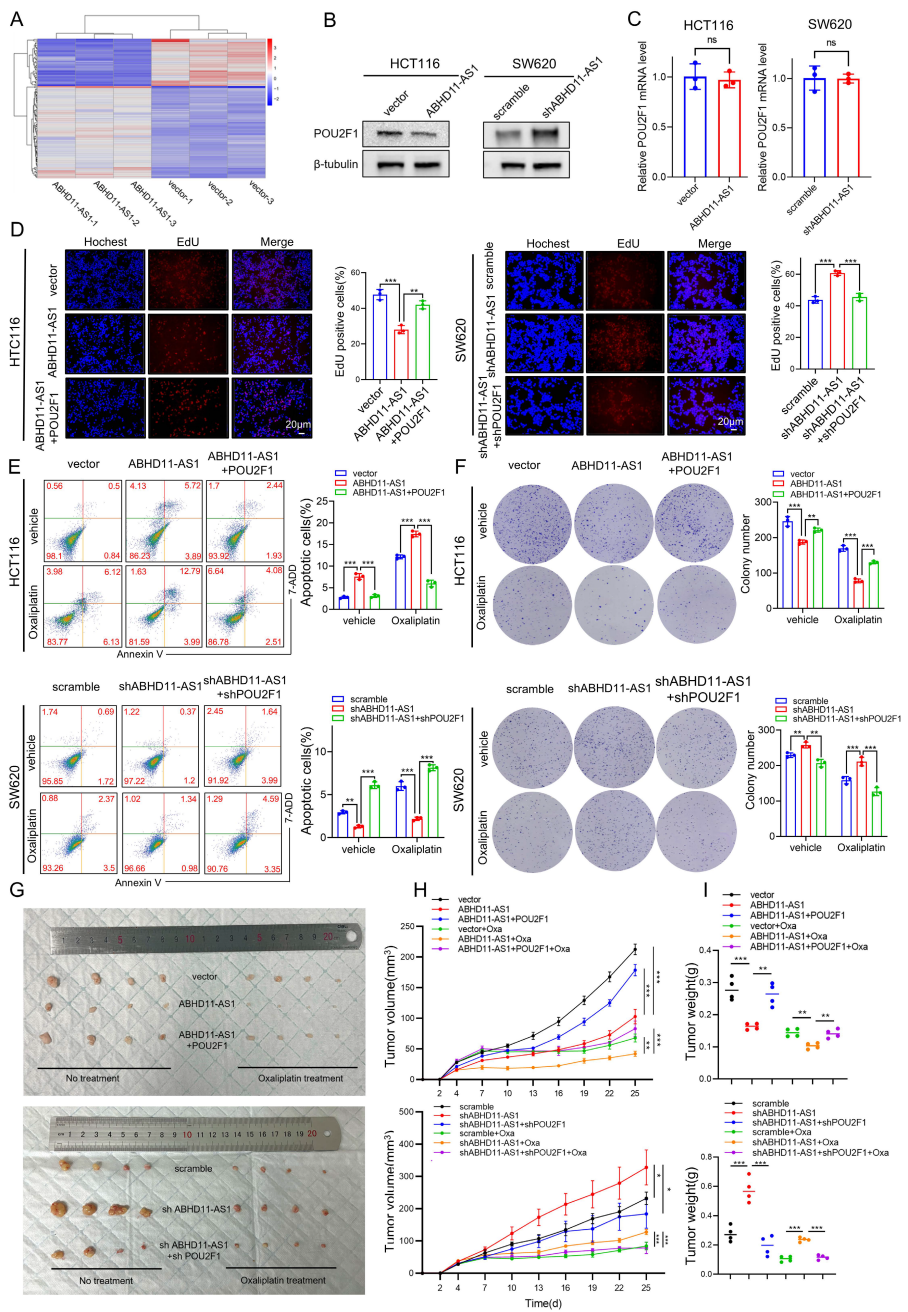
**POU2F1 acts as a critical downstream effector of ABHD11-AS1 mediated tumor suppression in colorectal cancer.** (A) Volcano plot illustrating differentially expressed proteins identified by proteomic analysis of HCT116 cells transfected with ABHD11-AS1 or vector. (B, C) Western blot and RT-qPCR analyses were performed to detect POU2F1 protein and mRNA expression levels following altered ABHD11-AS1 expression in HCT116 cells. (D) EdU incorporation assay was performed to evaluate CRC cell proliferation following POU2F1 rescue in HCT116 and SW620 cells with altered ABHD11-AS1 expression. (E, F) Flow cytometry-based apoptosis assay and colony formation assay were performed to evaluate the effect of restoring POU2F1 expression on oxaliplatin sensitivity in CRC cells with altered ABHD11-AS1 expression. (G-I) Subcutaneous xenograft tumor model in nude mice was used to further validate the regulatory role of POU2F1 in mediating the tumor-suppressive and chemosensitizing effects of ABHD11-AS1 *in vivo*. Data are presented as the mean ± SD of three independent experiments. Statistical analysis was performed using Student's t-test. Magnification, ×200; scale bar = 20 μm; ns, not significant; *P < 0.05; **P < 0.01; ***P < 0.001.

Our previous results showed that ABHD11-AS1 suppresses CRC progression and enhances oxaliplatin sensitivity *in vitro*, yet the underlying molecular mechanisms remain unclear. To elucidate the pathways mediating ABHD11-AS1 function, we conducted proteomic profiling in HCT116 cells transfected with either an ABHD11-AS1 overexpression construct or control vector (Figure 3A). Proteomic analysis identified 89 upregulated and 50 downregulated proteins in the ABHD11-AS1 overexpression group (|log₂FC| > 0.585, *P* < 0.05; Table S4). Notably, the expression of POU2F1 was significantly reduced in cells overexpressing ABHD11-AS1 compared with the control group (Supplementary Figure S2D). Western blot analysis confirmed that ABHD11-AS1 overexpression reduced POU2F1 protein levels in HCT116 cells, whereas its knockdown markedly increased POU2F1 expression. These results are consistent with the proteomic data (Figure 3B), in addition, we have found that ABHD11-AS1 have no significant impact on POU2F1 mRNA expression (Figure 3C). In our previous study, we showed that POU2F1 is upregulated in CRC and promotes tumor growth and oxaliplatin resistance by directly activating ALDOA transcription via promoter binding 33. These findings suggest that POU2F1 functions as a downstream effector of ABHD11-AS1, mediating its tumor-suppressive and chemosensitizing effects in CRC.

To determine whether ABHD11-AS1 regulates CRC progression and oxaliplatin sensitivity via downregulation of POU2F1, we restored POU2F1 expression in ABHD11-AS1-overexpressing CRC cells and, conversely, silenced POU2F1 in cells with ABHD11-AS1 knockdown (Supplementary Figure S3A). EdU assay showed POU2F1 reversed induced proliferation inhibition (Figure 3D). CCK8 assay and colony formation assay get the same results (Supplementary Figure S3B-C). Wound healing and Transwell assays corroborated these results, demonstrating that POU2F1 overexpression restored, while its silencing suppressed, the ABHD11-AS1-mediated effects on cell migration and invasion (Supplementary Figure S3D-G). Furthermore, flow cytometry and colony formation assays revealed that POU2F1 overexpression reversed the increased oxaliplatin sensitivity induced by ABHD11-AS1 overexpression, whereas POU2F1 silencing restored drug sensitivity in ABHD11-AS1-deficient cells (Figure 3E-F). To assess the role of POU2F1 in ABHD11-AS1-mediated tumor suppression and oxaliplatin sensitivity, a subcutaneous xenograft model was established. The results showed that POU2F1 overexpression attenuated the tumor growth inhibition and enhanced chemosensitivity induced by ABHD11-AS1 (Figure 3G-I). Taken together, these findings indicate that POU2F1 functions as a key downstream effector of ABHD11-AS1, mediating its tumor-suppressive activity and modulation of oxaliplatin sensitivity in CRC cells.

### ABHD11-AS1 inhibits USP18-mediated deubiquitination of POU2F1

**Figure 4:**
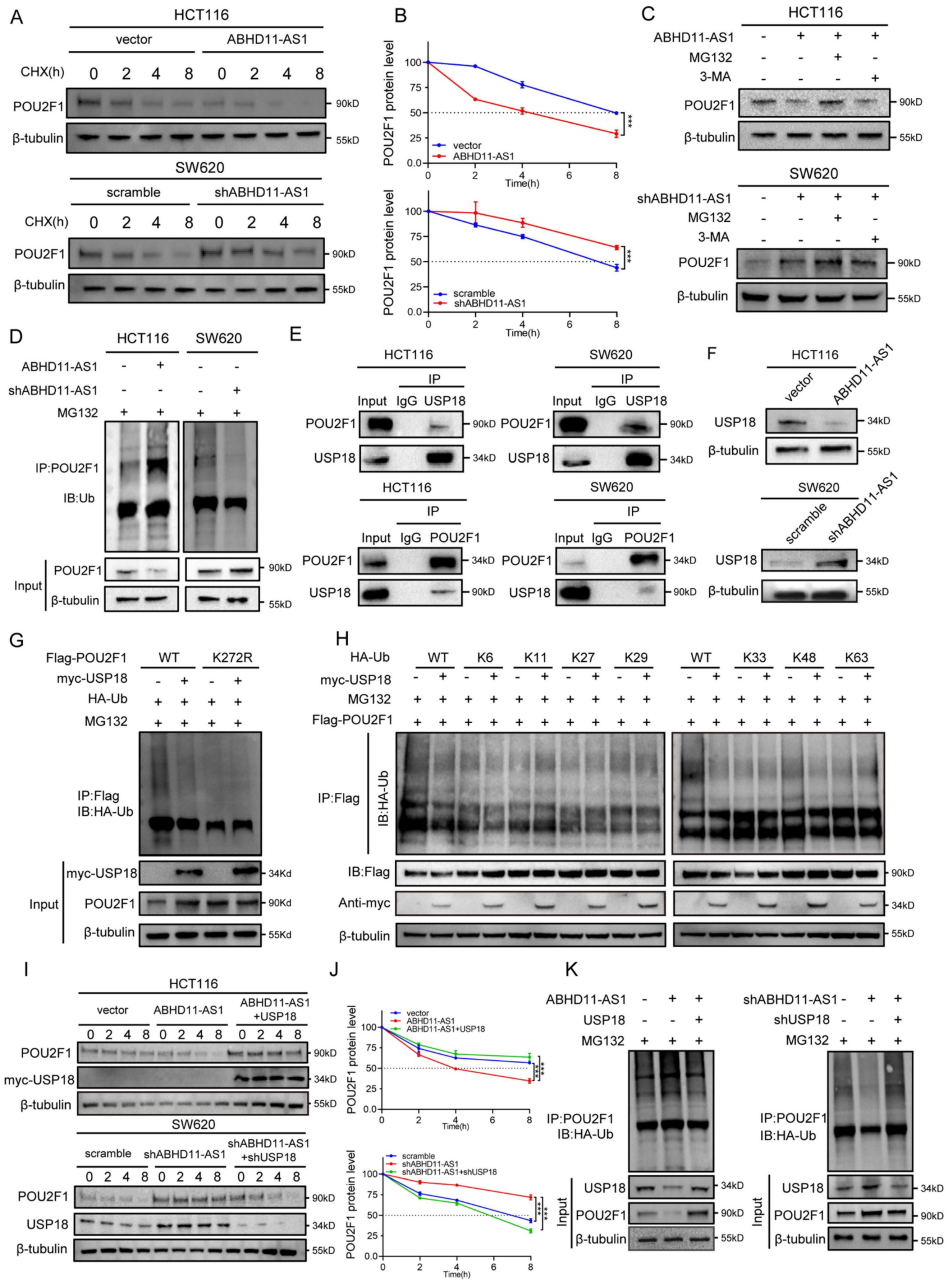
** ABHD11-AS1 inhibits USP18-mediated deubiquitination of POU2F1.** (A, B) CHX chase assay was performed to assess POU2F1 protein stability in HCT116 cells overexpressing ABHD11-AS1 and in SW620 cells with ABHD11-AS1 knockdown. (C) CRC cells were treated with MG132 or 3-MA to determine the ABHD11-AS1-regulated POU2F1 degradation pathway by western blotting. (D) Western blot analysis was performed to assess POU2F1 ubiquitination in HCT116 and SW620 cells with altered ABHD11-AS1 expression. (E) Co-IP assays were performed to assess the interaction between endogenous POU2F1 and USP18 in CRC cells. (F) Western blot analysis was conducted to examine USP18 protein levels following ABHD11-AS1 overexpression or knockdown in CRC cells. (G) Ubiquitination assays were performed using wild-type and K272-mutant POU2F1 constructs to identify the lysine residue targeted by USP18. (H) Ubiquitination assays with mutant ubiquitin constructs (K6, K11, K27, K29, K33, K48, and K63) were performed to determine the specific ubiquitin linkage removed by USP18. (I-K) Functional rescue experiments were performed to assess the role of USP18 in regulating POU2F1 stability and ubiquitination in CRC cells with altered ABHD11-AS1 expression. Data are presented as the mean ± SD of three independent experiments. *P < 0.05; **P < 0.01; ***P < 0.001.

Our previous findings revealed that ABHD11-AS1 downregulates POU2F1 protein levels without altering its mRNA expression, suggesting a post-transcriptional regulatory mechanism that may involve modulation of POU2F1 protein stability (Figure 3B-C). To evaluate the impact of ABHD11-AS1 on POU2F1 protein stability, HCT116 cells overexpressing ABHD11-AS1 were treated with the protein synthesis inhibitor cycloheximide (CHX). Overexpression of ABHD11-AS1 markedly reduced the half-life of POU2F1 protein. Conversely, knockdown of ABHD11-AS1 in SW620 cells led to prolonged POU2F1 protein stability (Figure 4A-B). These findings suggest that ABHD11-AS1 negatively regulates POU2F1 protein stability.

The ubiquitin-proteasome system (UPS) and the autophagy-lysosome pathway (ALP) constitute the two major protein degradation mechanisms in eukaryotic cells 50, 51. To investigate the mechanism underlying ABHD11-AS1-mediated POU2F1 degradation, CRC cells were treated with the proteasome inhibitor MG132 or the autophagy inhibitor 3-methyladenine (3-MA). MG132 effectively prevented ABHD11-AS1-induced degradation of POU2F1, whereas 3-MA had no appreciable effect (Figure 4C), indicating that ABHD11-AS1 promotes POU2F1 degradation primarily via the ubiquitin-proteasome system. In line with this, ubiquitination assays demonstrated that ABHD11-AS1 overexpression markedly enhanced POU2F1 ubiquitination, while its knockdown led to a substantial reduction in ubiquitination levels (Figure 4D). Taken together, these findings indicate that ABHD11-AS1 exerts its tumor-suppressive function, at least in part, by facilitating UPS-mediated degradation of POU2F1.

Ubiquitination is a key post-translational modification that regulates protein degradation, maintained by a dynamic balance between E3 ubiquitin ligases and deubiquitinating enzymes (DUBs) 52, 53. To further elucidate the molecular mechanism by which ABHD11-AS1 regulates POU2F1 ubiquitination, we analysed proteomic profiles of ABHD11-AS1-overexpressing cells, with a focus on ubiquitin-related enzymes. This analysis identified three DUBs with significantly altered expression (Supplementary Figure S4A). Among them, USP18 and USP11 were markedly downregulated and selected for further investigation as potential regulators. Co-immunoprecipitation assays revealed that endogenous USP18, but not USP11, interacts with POU2F1, suggesting a specific association (Figure 4E; Supplementary Figure S4B). Consistent with the proteomic data, Western blot analysis confirmed that ABHD11-AS1 overexpression reduced USP18 protein levels, whereas ABHD11-AS1 knockdown increased USP18 expression (Figure 4F). These results suggest that ABHD11-AS1 may regulate POU2F1 ubiquitination by modulating USP18 expression.

USP18 is a member of the DUB family, and previous studies have reported that USP18 plays an important role in CRC 54, 55. To investigate the relevance of USP18 in CRC, we analysed mRNA expression profiles from TCGA. USP18 expression was significantly upregulated in CRC tissues compared with normal tissues (Supplementary Figure S4C-D), a finding further validated by immunohistochemistry (Supplementary Figure S4E). To assess whether USP18 functions as the key DUB mediating ABHD11-AS1-dependent degradation of POU2F1, we generated a USP18 overexpression construct and designed two independent shRNAs targeting USP18. The efficiency of overexpression and knockdown was confirmed by qRT-PCR. Among the two candidates, shUSP18-2 exhibited the highest knockdown efficiency and was selected for subsequent experiments (Supplementary Figure S4F-G). Functional assays demonstrated that USP18 overexpression promoted HCT116 cell proliferation and colony formation, and this effect was partially reversed by POU2F1 knockdown. Conversely, USP18 knockdown inhibited proliferation and colony formation in SW620 cells, and this suppressive effect could be partially rescued by re-expression of POU2F1 (Supplementary Figure S5A-E).

Given that USP18 belongs to the USP family of deubiquitinating enzymes, we hypothesised that it may regulate POU2F1 degradation via the ubiquitin-proteasome system. Western blot analysis showed that USP18 overexpression increased POU2F1 protein levels without significantly affecting its mRNA expression, suggesting a post-translational mode of regulation (Figure 4F; Supplementary Figure S5F). CHX chase assays further confirmed that USP18 stabilizes POU2F1 at the post-translational level. USP18 overexpression prolonged the half-life of POU2F1, whereas USP18 knockdown shortened it (Supplementary Figure S5G). Consistently, ubiquitination assays showed that USP18 overexpression reduced POU2F1 ubiquitination, while USP18 knockdown enhanced its ubiquitination (Supplementary Figure S5H), indicating that USP18 stabilizes POU2F1 by removing its polyubiquitin chains.

As K272 has been reported to be a key ubiquitination site on POU2F1, we sought to determine whether USP18 directly mediates its deubiquitination 43. Ubiquitination assays using wild-type and K272-mutant POU2F1 constructs confirmed that K272 is the key residue deubiquitinated by USP18 (Figure 4G). To determine the ubiquitin linkage specificity of USP18, we used mutant ubiquitin constructs (K6, K11, K27, K29, K33, K48, and K63) in additional ubiquitination assays. The results showed that USP18 primarily removed K48-linked polyubiquitin chains from POU2F1, a classical signal for proteasomal degradation (Figure 4H). Finally, in HCT116 cells overexpressing ABHD11-AS1, reintroduction of USP18 restored POU2F1 stability and decreased its ubiquitination level. Conversely, USP18 knockdown in ABHD11-AS1-depleted SW620 cells reduced the half-life of POU2F1 and increased its ubiquitination (Figure 4I-K). Collectively, these findings demonstrate that ABHD11-AS1 promotes the degradation of POU2F1 by inhibiting USP18-mediated K48-linked deubiquitination, thereby suppressing CRC progression.

### ABHD11-AS1 interacts with EIF4E

**Figure 5:**
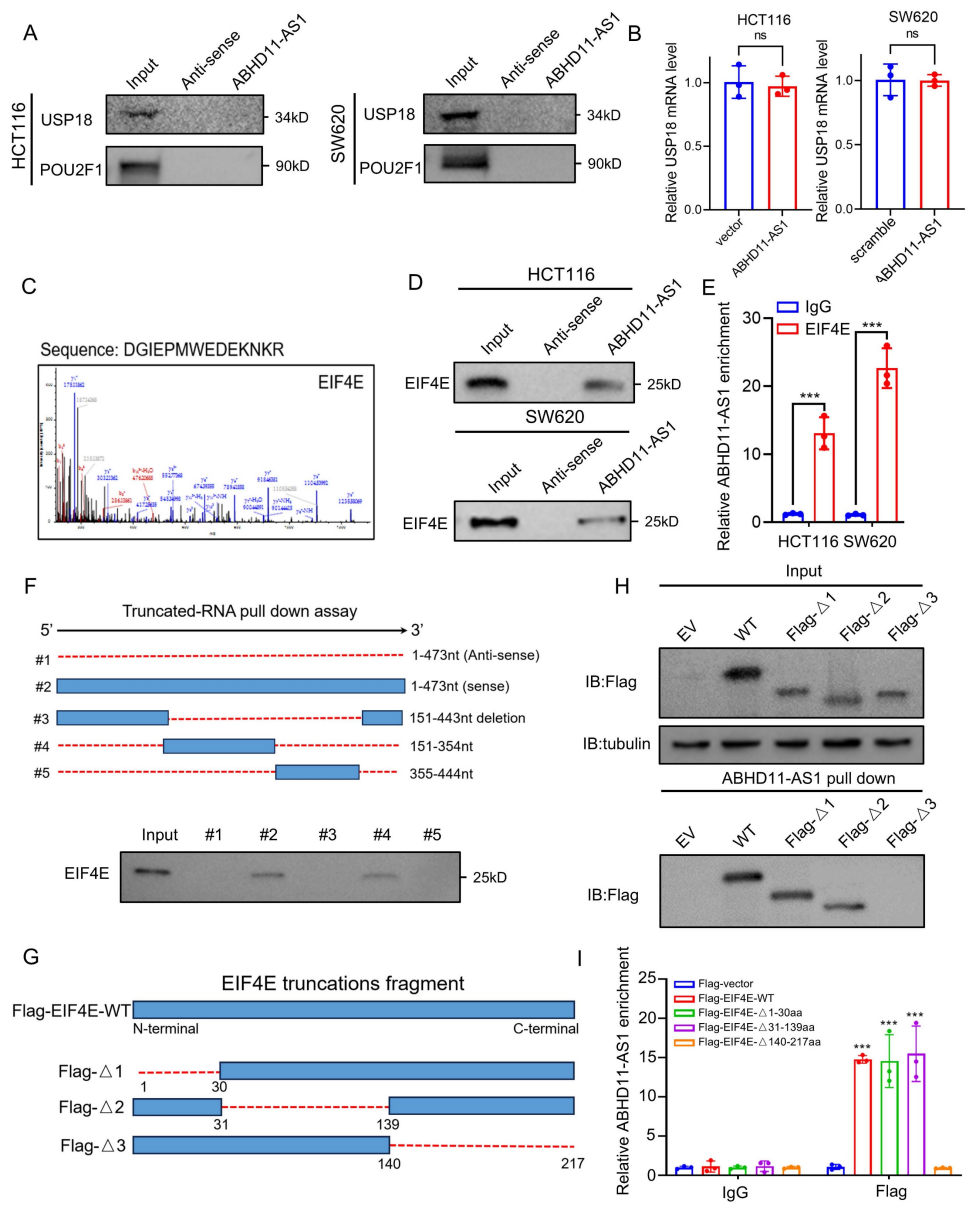
** ABHD11-AS1 interacts with EIF4E.** (A) RNA pulldown assays were performed to assess whether ABHD11-AS1 functions as a molecular scaffold facilitating interactions between USP18 and POU2F1. (B) Western blot and qRT-PCR analyses were conducted to evaluate the effects of ABHD11-AS1 expression on USP18 protein levels, mRNA expression, and protein stability. (C) Proteomic interactor analysis was performed to identify ABHD11-AS1-associated proteins. EIF4E was identified as a interactor and confirmed by MS/MS spectrum analysis. (D) RNA pulldown assays were conducted to validate the direct interaction between ABHD11-AS1 and EIF4E. (E) RIP followed by qRT-PCR was performed using EIF4E antibodies or IgG controls to confirm the association between ABHD11-AS1 and EIF4E. (F) RNA pulldown assays using full-length and truncated ABHD11-AS1 fragments were performed to map the EIF4E-binding region of ABHD11-AS1. (G, H) FLAG-tagged wild-type and truncated EIF4E constructs (Δ1: residues 1-30 deletion; Δ2: residues 31-139 deletion; Δ3: residues 140-217 deletion) were generated to define the EIF4E domain required for ABHD11-AS1 binding; RNA pulldown assays were performed to assess the binding capacity of ABHD11-AS1 with wild-type and truncated EIF4E constructs. (I) RIP assays were conducted to confirm that the Δ3 EIF4E fragment lacks ABHD11-AS1-binding capacity. Data are presented as the mean ± SD of three independent experiments. ns, not significant; ***P < 0.001.

It has been reported that certain non-coding RNAs can exert biological functions through encoding small peptides 56, 57. To assess the coding potential of ABHD11-AS1, we used two independent bioinformatic tools: the Coding Potential Calculator 2 (CPC2) (http://cpc2.gao-lab.org/) and the lncRNA cancer database lnCAR 58, 59. Both analyses consistently indicated that ABHD11-AS1 lacks protein-coding potential (Supplementary Figure S6A-B). Emerging evidence indicates that lncRNAs can act as molecular scaffolds to orchestrate the dynamic interplay between E3 ubiquitin ligases or DUBs and their substrate proteins, thereby modulating protein stability and function 60-62. To investigate whether ABHD11-AS1 functions as a molecular scaffold facilitating the interaction between USP18 and POU2F1, we first performed RNA pull-down assays. Neither USP18 nor POU2F1 was detected in the ABHD11-AS1-associated complex, excluding the possibility of a direct scaffold role for ABHD11-AS1 (Figure 5A). Interestingly, although ABHD11-AS1 markedly influenced USP18 protein expression, it had no detectable effect on USP18 mRNA levels or protein stability, suggesting that ABHD11-AS1 regulates USP18 expression through an alternative, post-transcriptional mechanism (Figure 5B; Supplementary Figure S6C). Therefore, we hypothesized that ABHD11-AS1 may regulate USP18 expression at the translational level. Sucrose gradient fractionation showed that, compared with the control group, knockdown of ABHD11-AS1 shifted the USP18 mRNA peak toward higher-density/heavier fractions, suggesting enhanced association with polysomes and increased translational activity (Supplementary Figure S6D). To further investigate the mechanism by which ABHD11-AS1 regulates USP18 expression, we performed SDS-PAGE followed by Coomassie Brilliant Blue staining of proteins retrieved from the ABHD11-AS1 pull-down complex (Figure 5C; Supplementary Figure S6E-F). Subsequent mass spectrometry analysis identified 86 putative ABHD11-AS1-interacting proteins, including two factors involved in translational regulation (Supplementary Figure S6G and Supplementary Table S5). Notably, the top-ranking candidate was eukaryotic translation initiation factor 4E (EIF4E), a central component of the translation initiation complex that controls mRNA translation and is implicated in cancer progression (Figure 5C) 63. Based on this finding, we hypothesized that ABHD11-AS1 may regulate USP18 translation through interaction with EIF4E. Supporting this, Western blot analysis confirmed that EIF4E was enriched in the ABHD11-AS1 pull-down complex, but not in the antisense control group (Figure 5D). Consistently, RIP combined with qRT-PCR showed that EIF4E antibody precipitated a significant amount of ABHD11-AS1 transcripts, while the IgG control did not (Figure 5E), further suggesting a potential interaction between ABHD11-AS1 and EIF4E. To identify the specific regions mediating this interaction, we predicted the secondary structure of ABHD11-AS1 using ViennaRNA Web Services (Supplementary Figure S6H), and constructed a series of full-length and truncated ABHD11-AS1 mutants for pull-down assays. The results indicated that EIF4E binds to full-length ABHD11-AS1 and truncated fragment #4, but not to the antisense control, fragment #3, or fragment #5 (Figure 5F; Supplementary Figure S6I), suggesting that 151-354nt of ABHD11-AS1 are essential for EIF4E binding. To identify the EIF4E domain responsible for ABHD11-AS1 interaction, we generated FLAG-tagged wild-type and truncated EIF4E mutants: Δ1 (deletion of residues 1-30aa), Δ2 (31-139aa), and Δ3 (140-217aa) (Figure 5G). RNA pull-down assays showed that ABHD11-AS1 failed to bind the Δ3 mutant (Figure 5H), and RIP assays further confirmed that Δ3 EIF4E lacked binding affinity for ABHD11-AS1 (Figure 5I), indicating that amino acid residues 140-217 of EIF4E are critical for mediating this interaction. In summary, our findings demonstrate that the 151-354nt region of ABHD11-AS1 and the 140-217aa region of EIF4E are essential domains required for their interaction.

### ABHD11-AS1 disrupts EIF4E phase separation to suppress USP18 expression

Further investigation revealed that changes in ABHD11-AS1 expression did not alter EIF4E protein levels (Supplementary Figure S7A), suggesting that ABHD11-AS1 regulates EIF4E through alternative mechanisms. Phase separation plays a critical role in translational control by enabling the spatial compartmentalization of translation initiation factors, ribosomes, and mRNAs into membraneless condensates. These condensates facilitate selective and efficient translation. For instance, FXR1 forms phase-separated condensates that enrich specific mRNAs and recruit the translational machinery via interaction with EIF4G3, thereby activating translationally repressed mRNA pools and promoting protein synthesis 41. In cancer, dysregulated phase separation can reprogram selective mRNA translation, driving the preferential synthesis of oncogenic proteins and contributing to malignant progression. For example, during the blast crisis phase of chronic myeloid leukemia, PABPC1 undergoes phase separation to promote the selective translation of BCR-ABL1 and its downstream effectors, thereby facilitating disease advancement and conferring resistance to tyrosine kinase inhibitors 42.

**Figure 6:**
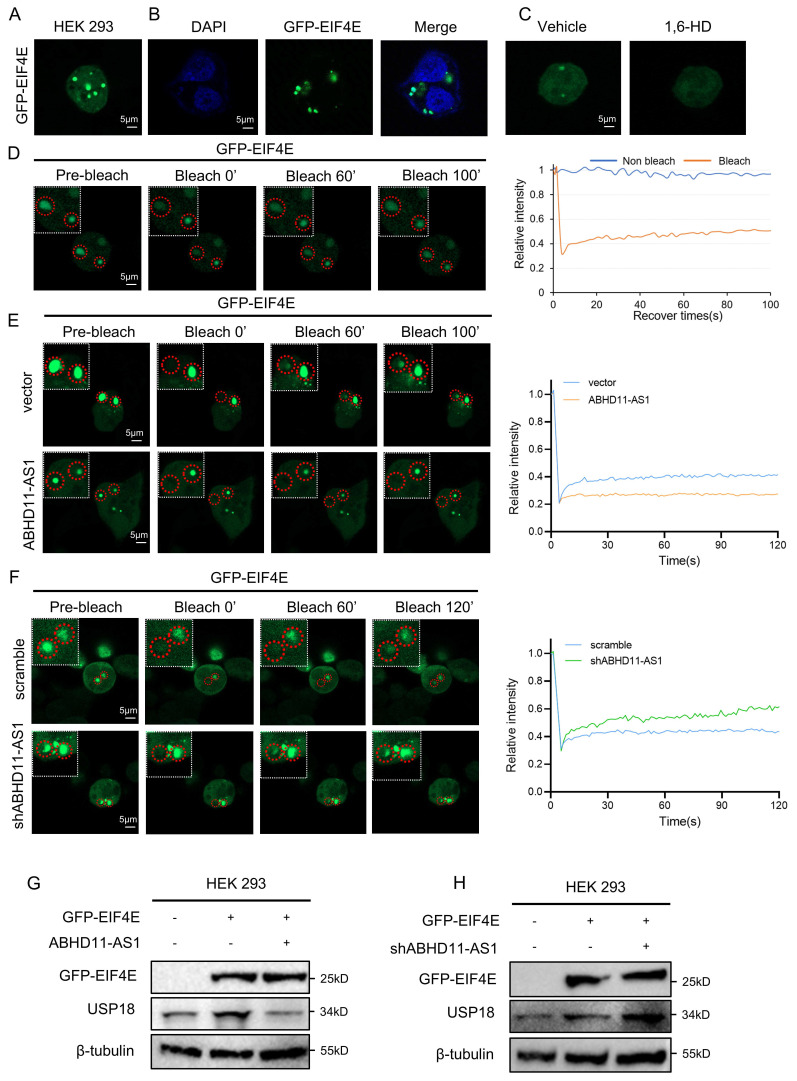
** ABHD11-AS1 disrupts EIF4E phase separation to suppress USP18 expression.** (A, B) GFP-tagged EIF4E was expressed in HEK293 cells to assess condensate formation by fluorescence microscopy. (C) Cells were treated with 1% 1,6-HD to evaluate the effect of phase separation disruption on EIF4E condensate formation. (D) FRAP analysis was performed to assess the dynamic properties of EIF4E condensates. (E, F) FRAP analysis was conducted to evaluate the effect of ABHD11-AS1 overexpression or knockdown on EIF4E condensate recovery dynamics. (G, H) Western blot analysis was performed to assess the effects of ABHD11-AS1 overexpression or knockdown on USP18 protein expression. Scale bar, 5μm (confocal microscopy).

To assess the phase separation potential of EIF4E, we first analyzed its intrinsically disordered regions (IDRs) using the PONDR (Predictor of Natural Disordered Regions) algorithm, which revealed distinct IDR segments within the EIF4E protein (Supplementary Figure S7B). We next generated a GFP-tagged EIF4E construct and expressed it in HEK293 cells. Fluorescence microscopy revealed the formation of discrete punctate condensates, a characteristic feature of phase separation (Figure 6A-B). Treatment with the classical phase separation-disrupting agent 1,6-hexanediol (1,6-HD) markedly diminished EIF4E condensates, confirming their liquid-like properties (Figure 6C). Furthermore, fluorescence recovery after photobleaching (FRAP) demonstrated that EIF4E puncta exhibited dynamic molecular exchange, providing further evidence of their liquid-like, phase-separated nature (Figure 6D). Consistent with these results, we observed similar EIF4E puncta in CRC cell lines, which were disrupted by 1,6-HD and exhibited dynamic recovery in FRAP assays (Supplementary Figure S7C-D). Meanwhile, we generated a series of GFP-tagged EIF4E deletion mutants and found that their ability to form punctate condensates was markedly reduced or nearly abolished, indicating that all three regions contribute critically to EIF4E condensate formation (Supplementary Figure S7E). Intriguingly, 1,6-HD treatment not only disrupted EIF4E condensates but also significantly reduced USP18 protein expression (Supplementary Figure S7F), suggesting that EIF4E phase separation may facilitate USP18 translation.

To further investigate the role of ABHD11-AS1 in this process, we examined its effect on EIF4E phase separation. In contrast, overexpression of ABHD11-AS1 markedly impaired the fluorescence recovery of EIF4E condensates after photobleaching, accompanied by reduced puncta size and fluorescence intensity. Conversely, knockdown of ABHD11-AS1 promoted the reformation of EIF4E condensates and resulted in larger puncta with increased fluorescence intensity (Figure 6E-F; Supplementary Figure S7G-H). These findings suggest that ABHD11-AS1 negatively regulates the dynamic process of EIF4E phase separation. Consistent with these findings, Western blot analysis revealed that ABHD11-AS1 overexpression reduced USP18 protein levels, whereas its knockdown led to increased USP18 expression (Figure 6G-H). Together, these results indicate that ABHD11-AS1 suppresses USP18 expression by disrupting the phase separation behavior of EIF4E, thereby contributing to its tumor-suppressive function.

Finally, we examined the expression of ABHD11-AS1, POU2F1, USP18, and EIF4E in xenograft tumor sections from mice in different treatment groups. The results showed that the staining intensity signal of POU2F1 and USP18 was markedly decreased in the ABHD11-AS1 overexpression group, whereas both were significantly increased in the shABHD11-AS1 group (Supplementary Figure S8A). In contrast, EIF4E exhibited no obvious differences in overall expression among the groups. Collectively, these findings further support a negative correlation between ABHD11-AS1 and the expression of POU2F1 and USP18, consistent with our observations at the cellular level.

## Discussion

In recent years, transcriptomic studies have revealed that protein-coding mRNAs account for less than 2% of the human genome, with the vast majority of transcripts being ncRNAs 64. Among these, lncRNAs have emerged as key regulatory molecules in tumor biology, and several phase I and II clinical trials are currently underway to explore their therapeutic potential in cancer 65. Moreover, advances in high-throughput sequencing technologies have markedly accelerated the identification and functional annotation of lncRNAs. Accumulating evidence underscores their pivotal involvement in tumor initiation, progression, and therapeutic resistance 11.

These findings underscore the context-dependent roles of H19, which functions as an oncogene in some cancers, such as cutaneous squamous cell carcinoma, but acts as a tumor suppressor in others, including osteosarcoma via suppression of SNORA7A 66, 67. In lung cancer, H19 is upregulated and functions as a sponge for miR-200a, thereby promoting the expression of ZEB1 and ZEB2, which in turn enhances cell proliferation and metastasis 68. Conversely, another study on EGFR-mutant non-small cell lung cancer found that β-elemene enhances erlotinib sensitivity by inducing ferroptosis through H19 upregulation 69. LncRNA TINCR has been widely reported as an oncogenic factor that promotes the progression of multiple cancers, including cervical cancer, CRC, and hepatocellular carcinoma 70-72. However, in laryngeal squamous cell carcinoma, TINCR appears to function as a tumor suppressor by inhibiting cell proliferation and metastasis through modulation of the miR-210/APR2 axis 73. In CRC, loss of TINCR has been shown to promote EpCAM cleavage and nuclear translocation of EpICD, thereby activating the Wnt/β-catenin signalling pathway 74.

Similarly, PVT1 has been characterized as a tumor-associated lncRNA with oncogenic functions in multiple cancers 75, 76. Interestingly, because PVT1 and MYC are located within the same topologically associating domain, the PVT1 promoter can also act as a tumor-suppressive DNA regulatory element under specific conditions 77. LncRNA SNHG1 is involved in several tumor-related processes, including cell proliferation, metastasis, ferroptosis, and chemoresistance 78, 79. However, its role in gastric cancer remains controversial. One study reported that SNHG1 functions as a competing endogenous RNA for miR-195-5p, thereby promoting gastric cancer cell proliferation by upregulating YAP1 80; conversely, another study showed that SNHG1 suppresses cell invasion by modulating the SOCS2/JAK2/STAT signaling pathway 81. These conflicting findings may be attributed to inter-individual variability, tissue specificity, and intratumoral heterogeneity. Therefore, future tumor research should pay greater attention to these factors and incorporate precise molecular characterization into tumor classification and the development of personalized therapeutic strategies. In our study, ABHD11-AS1 was significantly downregulated in tumor tissues compared to matched normal tissues in both the TCGA-COAD and TCGA-READ datasets, consistent with our experimental evidence supporting its tumor-suppressive role. Notably, a previous study reported upregulation of ABHD11-AS1 in TCGA-COAD and -READ samples relative to normal tissues from the combined TCGA and GTEx datasets. This discrepancy may stem from differences in reference controls, as the GTEx dataset includes normal tissues from 54 distinct organ types, which may not be directly comparable to adjacent colorectal tissue 82, 83.

Ubiquitination is a critical post-translational modification governed primarily by E3 ubiquitin ligases and DUBs. It plays a central role in regulating protein stability and degradation, cell cycle progression, DNA repair, and signal transduction, and is increasingly recognised as a key contributor to tumorigenesis and cancer progression. Emerging evidence suggests that lncRNAs can modulate ubiquitination and deubiquitination through diverse mechanisms, thereby affecting the stability of target proteins and promoting tumor progression 84-86. In our study, overexpression of ABHD11-AS1 markedly reduced POU2F1 protein levels in CRC cells, whereas ABHD11-AS1 knockdown led to a significant increase, suggesting that ABHD11-AS1 may exert its tumor-suppressive function by modulating POU2F1 protein stability. Mechanistically, we found that ABHD11-AS1 suppresses the translation of USP18, a deubiquitinating enzyme known to stabilise POU2F1. Consequently, reduced USP18 levels impaired the deubiquitination of POU2F1, resulting in enhanced ubiquitination and proteasomal degradation. These findings uncover a previously unrecognised post-transcriptional regulatory axis through which ABHD11-AS1 inhibits CRC progression.

EIF4E is a central component of the eukaryotic translation initiation complex and functions as a key rate-limiting factor in the initiation of mRNA translation 87. Its primary function is to recognise the 7-methylguanosine (m⁷G) cap at the 5′ end of mRNAs, thereby facilitating the initiation of translation 88. Numerous studies have shown that EIF4E is frequently upregulated in diverse cancers, where it drives tumor progression by promoting proliferation, invasion, angiogenesis, and resistance to chemotherapy, and is correlated with unfavourable clinical outcomes 89, 90. In this study, mass spectrometry analysis identified EIF4E as a direct binding partner of ABHD11-AS1. Notably, modulation of ABHD11-AS1 levels did not alter the overall expression of EIF4E. Mechanistically, we demonstrated that ABHD11-AS1 impairs the phase separation capacity of EIF4E, thereby suppressing the expression of its downstream target, USP18. This, in turn, enhances the ubiquitination and proteasomal degradation of POU2F1, ultimately inhibiting CRC progression. These findings uncover a previously unrecognised mechanism by which ABHD11-AS1 exerts its tumor-suppressive function via disruption of EIF4E-mediated phase separation, underscoring the importance of RNA-protein interactions in modulating translation-associated phase separation and oncogenic signalling pathways.

In summary, this study identifies a previously unrecognised mechanism by which ABHD11-AS1 suppresses CRC progression through modulation of phase separation, thereby regulating downstream protein expression and stability. Our findings highlight the critical role of lncRNAs in spatial cellular compartmentalisation and offer broader insights into the regulatory architecture of tumor biology. Nonetheless, several limitations remain. First, the conclusions are primarily derived from *in vitro* and *in vivo* experimental models, and further validation in large, well-characterised clinical cohorts is warranted to substantiate the prognostic and therapeutic relevance of ABHD11-AS1 in CRC. Although we delineated the central axis by which ABHD11-AS1 suppresses USP18 translation and promotes POU2F1 degradation, it remains unclear whether ABHD11-AS1 engages additional oncogenic or tumor-suppressive pathways. Future studies should explore the development of nucleotide analogues or delivery platforms based on ABHD11-AS1, and assess their therapeutic efficacy—alone or in combination with oxaliplatin-across molecular subtypes of CRC, particularly in microsatellite instability-high (MSI-H) subgroups that exhibit poor responses to conventional chemotherapy. Moreover, investigating the functional roles and therapeutic potential of ABHD11-AS1 in other cancer types will be essential to evaluate its feasibility and universality as a candidate anticancer target.

## Conclusion

In conclusion, this study identifies ABHD11-AS1 as a tumor-suppressive long non-coding RNA that is downregulated in CRC and associated with poor patient prognosis. Functional assays demonstrated that ABHD11-AS1 inhibits CRC cell proliferation and migration and enhances sensitivity to oxaliplatin by downregulating POU2F1 protein levels. Mechanistically, ABHD11-AS1 promotes POU2F1 degradation by suppressing USP18-mediated deubiquitination, thereby reducing POU2F1 protein stability. Notably, ABHD11-AS1 directly interacts with EIF4E and disrupts its phase separation capacity, leading to reduced USP18 translation. This study uncovers a previously unrecognised mechanism by which ABHD11-AS1 regulates protein homeostasis through modulation of EIF4E-mediated phase separation, ultimately destabilising the oncogenic protein POU2F1. These findings not only expand our understanding of lncRNA-mediated control of phase separation and protein turnover but also position ABHD11-AS1 as a promising therapeutic target for overcoming chemoresistance and improving treatment outcomes in CRC.

## Supplementary Material

Supplementary figures and tables.

## Figures and Tables

**Figure 7:**
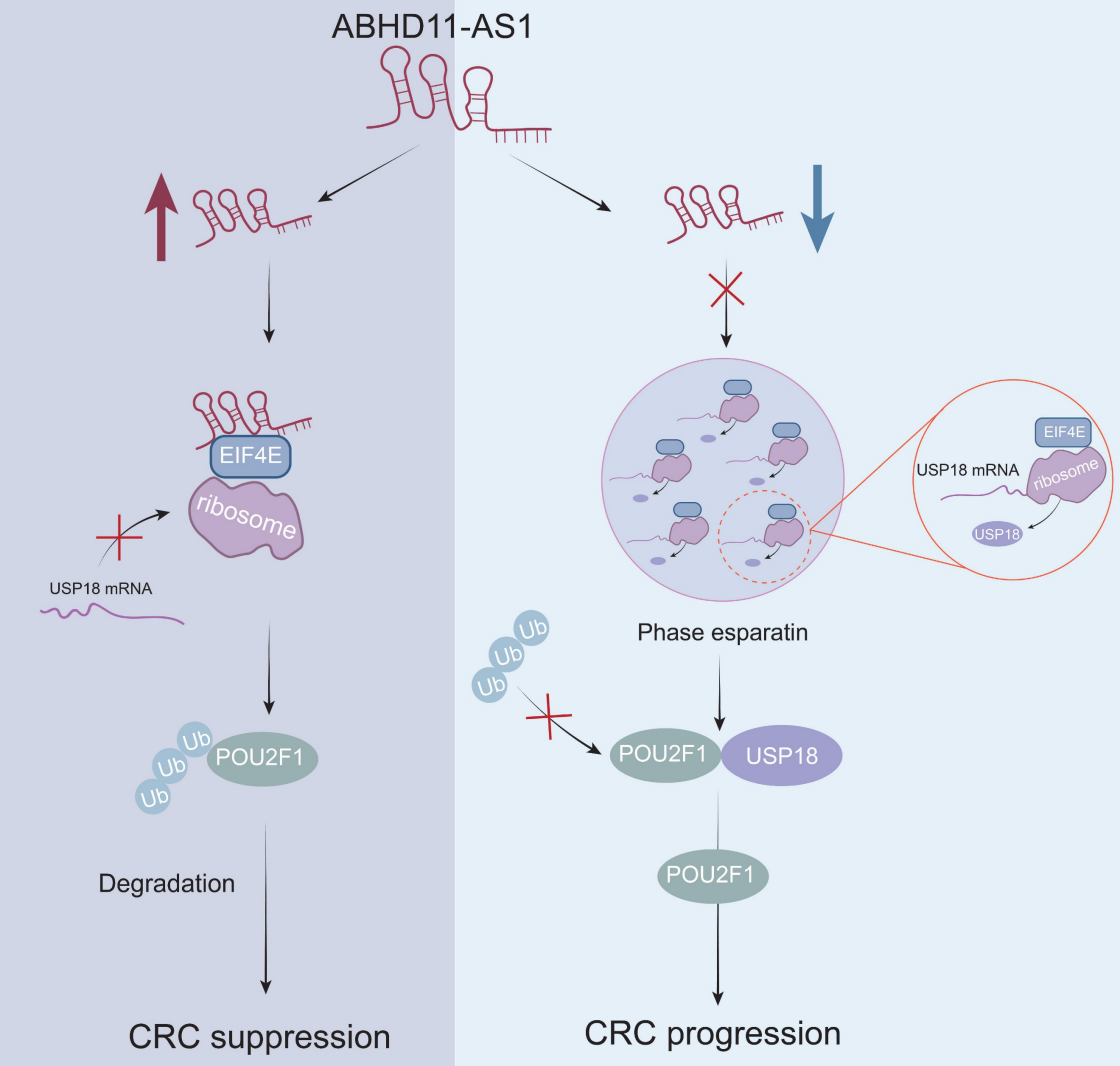
** ABHD11-AS1 suppresses colorectal cancer progression by disrupting EIF4E phase separation-mediated POU2F1 ubiquitination**.

## Data Availability

The relevant datasets have been provided in the supplementary materials.
