# Supplementary material for: ABHD11-AS1 Suppresses Colorectal Cancer Progression by Disrupting EIF4E-mediated POU2F1 Ubiquitination
Source: Int J Biol Sci. 2026 Apr 23;22(9):4765–83. doi: 10.7150/ijbs.125799 (PMC13182537; doi:10.7150/ijbs.125799)
Supplement: Supplementary file 1 — Supplementary figures and tables. [file ijbsv22p4765s1.pdf]

Supplementary Materials

Supplementary Figure S1

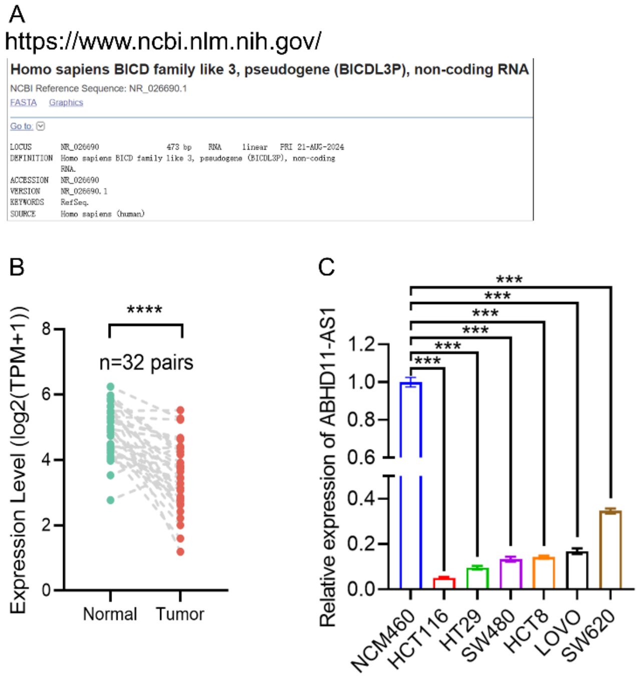

Supplementary Figure S1. Basic characterization of ABHD11-AS1.

(A) Detailed information on ABHD11-AS1 (also known as BICDL3P) obtained from the National Center for Biotechnology Information (NCBI) database (https://www.ncbi.nlm.nih.gov/).

(B) Analysis of ABHD11-AS1 expression in paired tumor and adjacent normal tissue samples using TCGA-COAD transcriptomic data.

(C) ABHD11-AS1 expression levels were measured by RT-qPCR in different CRC cell lines.

Supplementary Figure S2

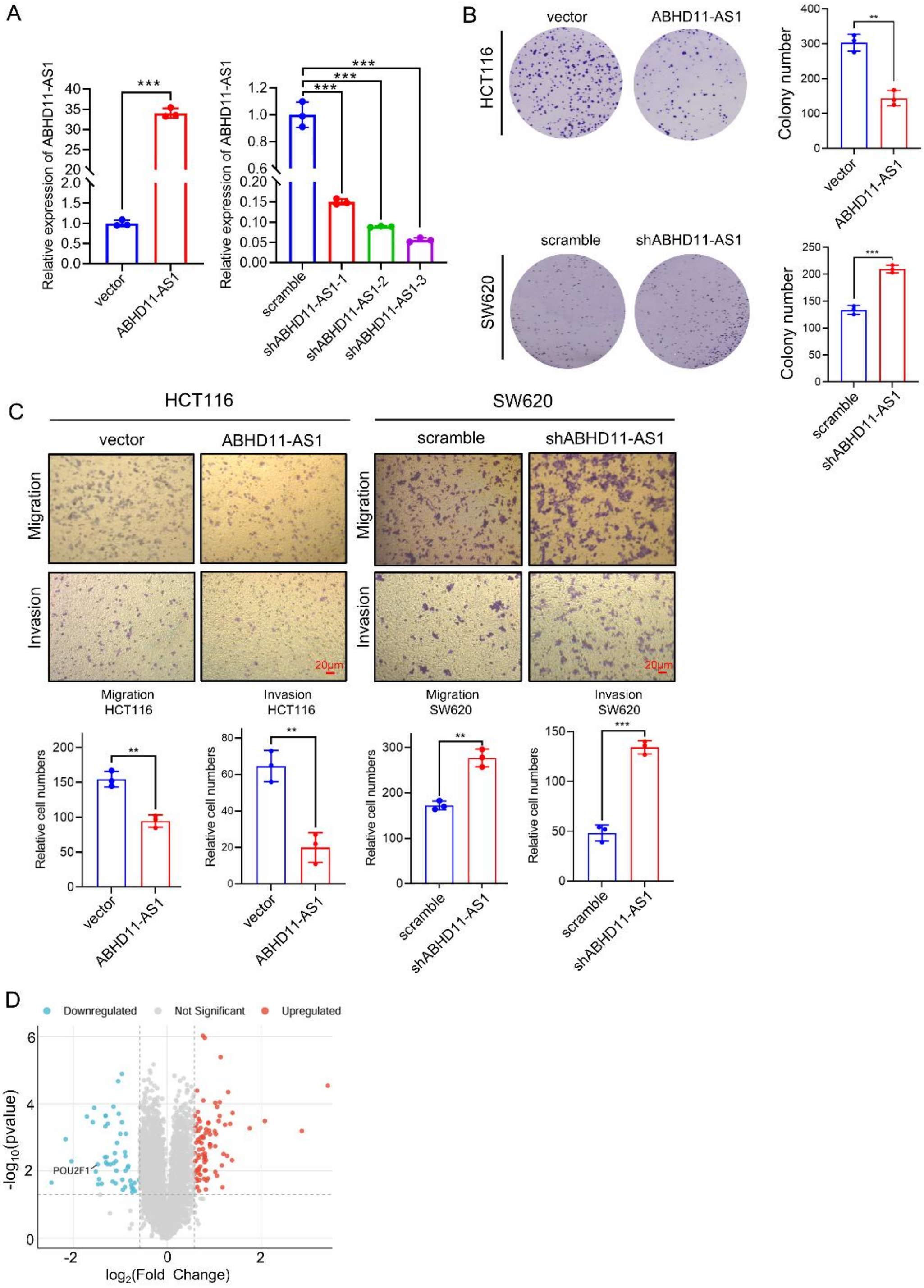

Supplementary Figure S2. ABHD11-AS1 suppresses colorectal cancer cell colony formation and migration.

(A) qRT-PCR was performed to confirm the efficiency of ABHD11-AS1 overexpression and knockdown in HCT116 and SW620 cells.

(B) Colony formation assay was performed to evaluate the clonogenic ability of CRC cells following altered expression of ABHD11-AS1 in HCT116 and SW620 cells.

(C) Transwell assay was performed to evaluate the migration and invasion abilities of

CRC cells following altered expression of ABHD11-AS1 in HCT116 and SW620 cells.
(D) Volcano plot illustrating the differential protein expression profile in HCT116 cells. POU2F1 is highlighted in the plot to indicate its altered expression.
Data are presented as the mean ± SD from three independent experiments.
Magnification, ×200; scale bar = 20 μm; ***P* < 0.01; ****P* < 0.001;

Supplementary Figure S3

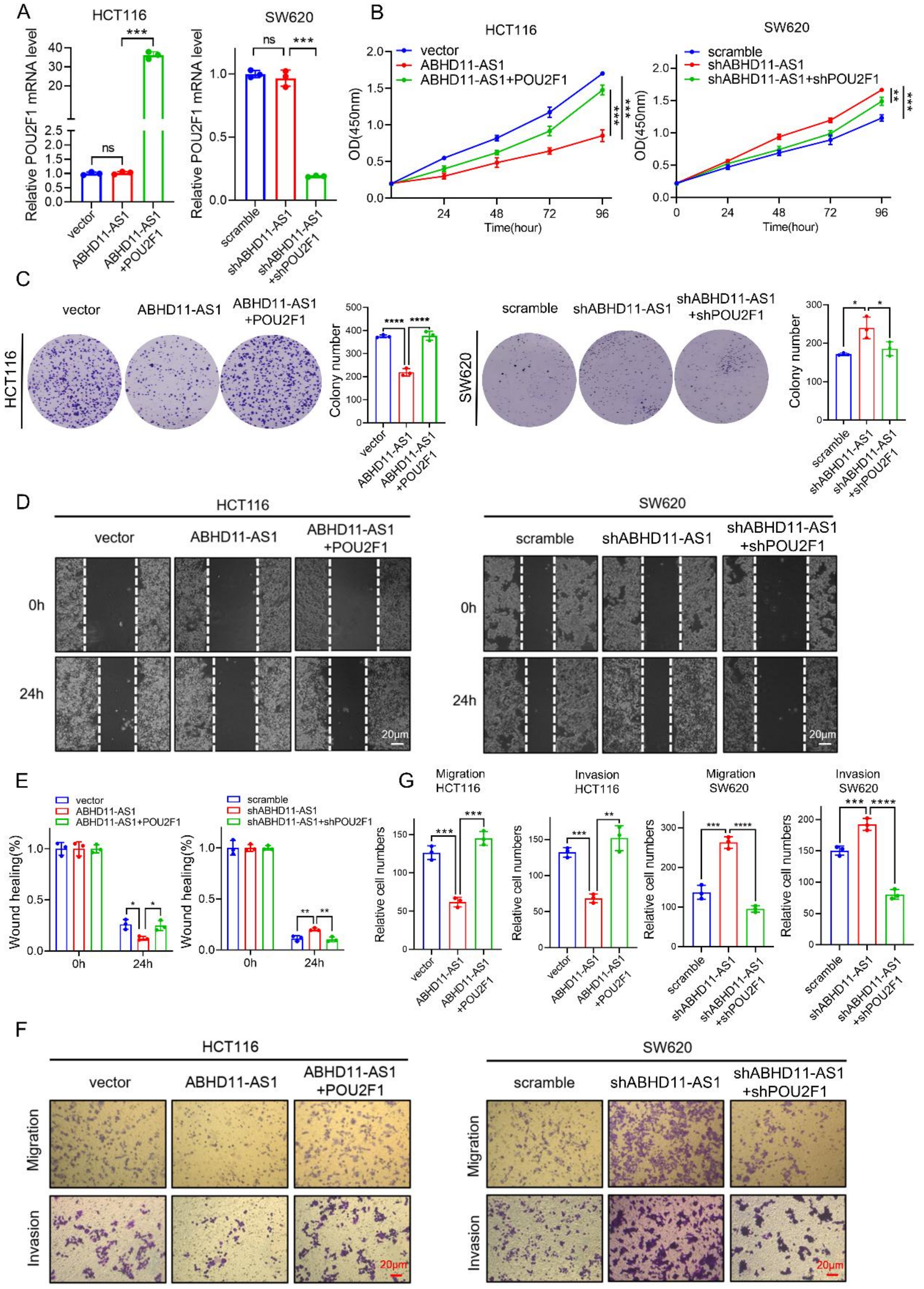

Supplementary Figure S3. POU2F1 mediates the effects of ABHD11-AS1 on CRC cell proliferation, migration, and invasion.

(A) RT-qPCR demonstrated the successful rescue of POU2F1 expression in HCT116 cells and SW620 cells with altered ABHD11-AS1 expression.

(B, C) CCK-8 assay and colony formation assay were performed to evaluate CRC cell

proliferation following POU2F1 rescue in HCT116 and SW620 cells with altered ABHD11-AS1 expression.

(D, E) Wound healing assay and Transwell assay were performed to evaluate CRC migration and invasion following POU2F1 rescue in HCT116 and SW620 cells with altered ABHD11-AS1 expression. Data are presented as the mean ± SD of three independent experiments. Magnification, ×200; scale bar = 20μm; ns, not significant; *$P < 0.05$; **$P < 0.01$; ***$P < 0.001$.

Supplementary Figure S4

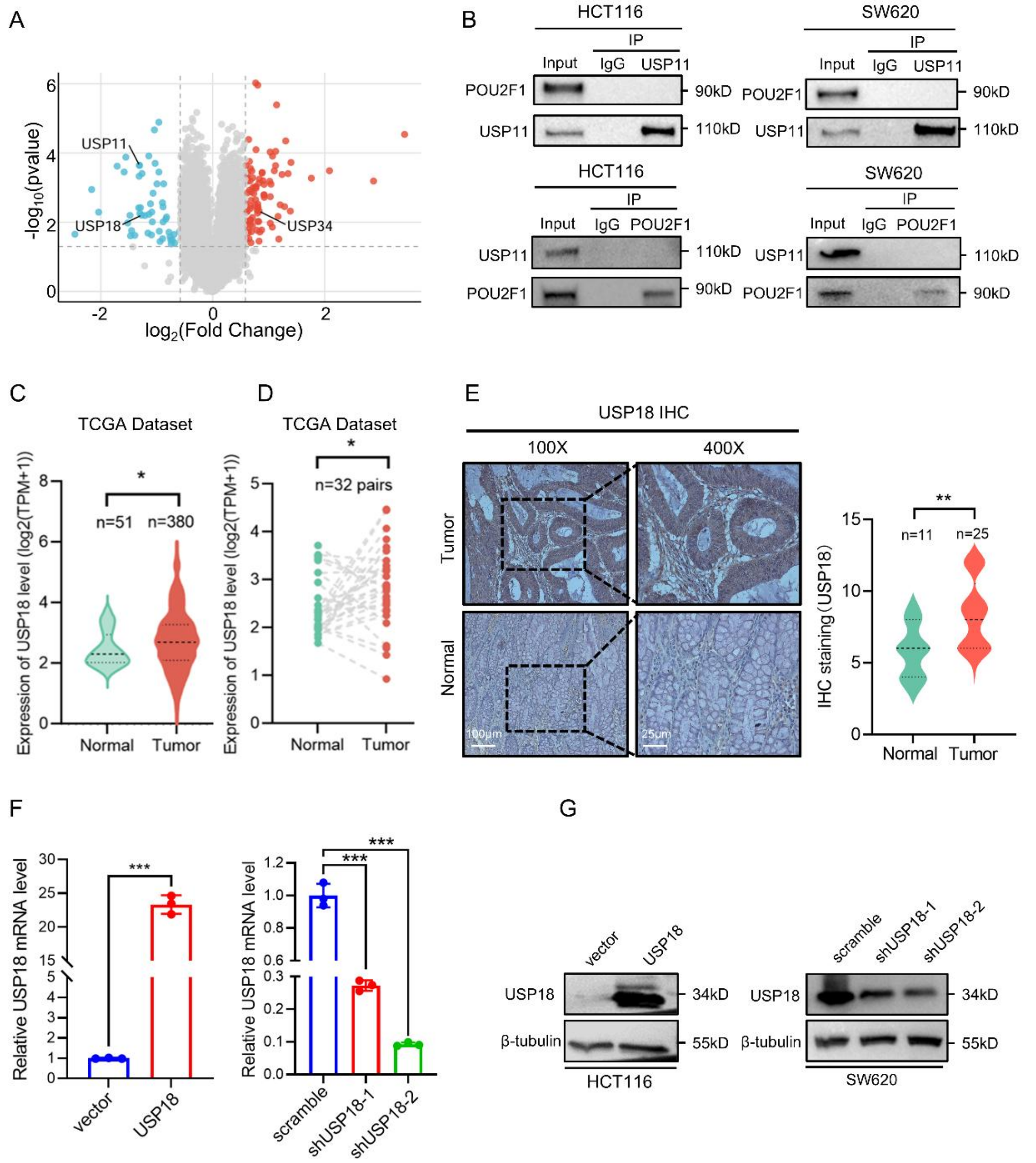

Supplementary Figure S4. Identification USP18 as the key DUB involving in ABHD11-AS1-regulated POU2F1 degradation.

(A) Proteomic analysis was performed to identify ABHD11-AS1-induced changes in ubiquitination-related enzymes, including USP11, USP18 and USP34.

(B) Co-IP assays were performed to assess the interaction between endogenous POU2F1 and USP11 in CRC cells.

(C, D) USP18 mRNA expression was analyzed in CRC tissues and adjacent normal tissues using TCGA datasets.

(E) IHC analysis was conducted to evaluate USP18 protein expression in CRC tissues and adjacent normal tissues.

(F) USP18 overexpression plasmid and three shRNAs targeting USP18 were generated, and their efficacy was validated by qRT-PCR analysis.

Data are presented as the mean ± SD of three independent experiments. *$P < 0.05$; **P

< 0.01; ***P < 0.001.

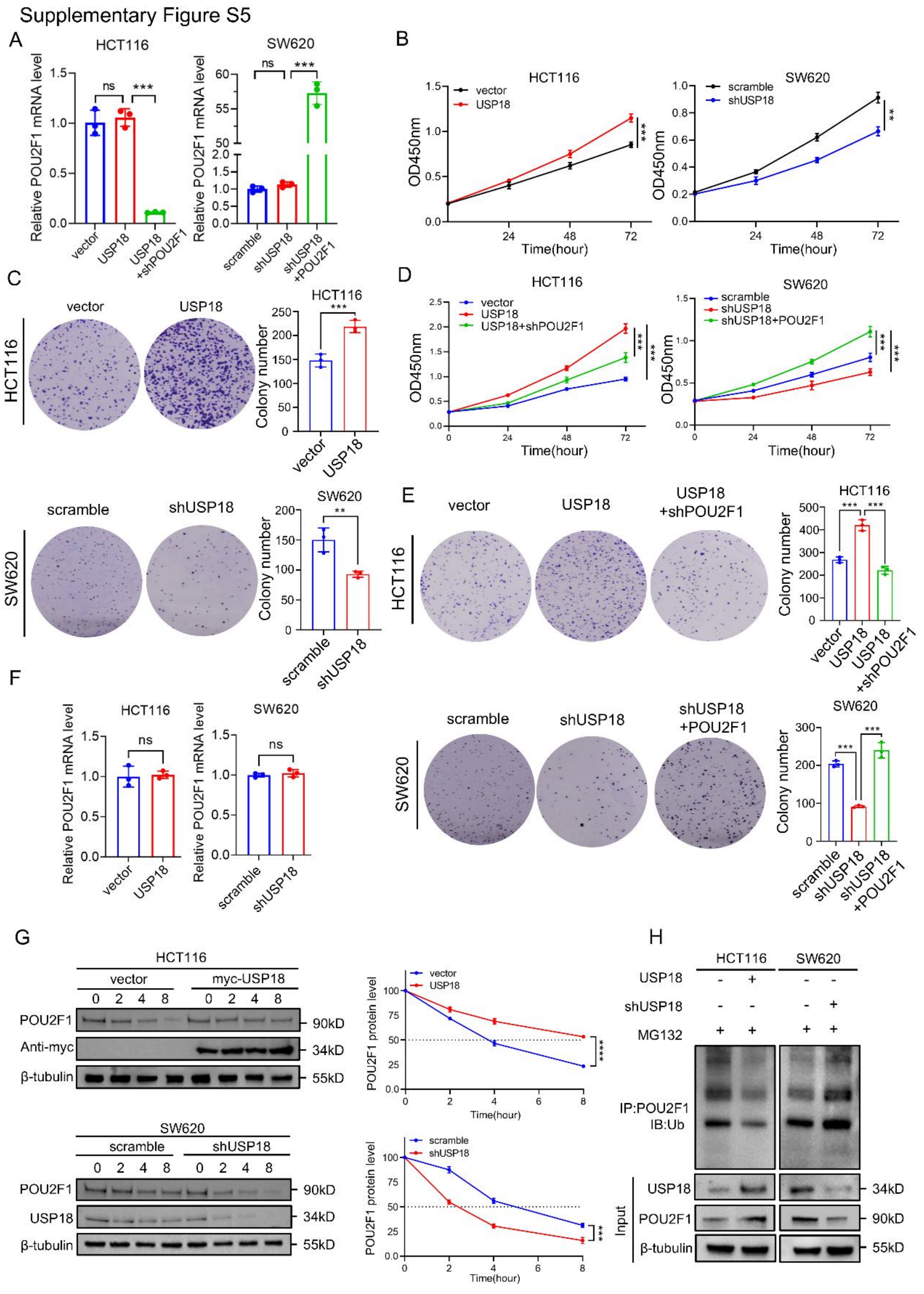

Supplementary Figure S5. USP18 promotes CRC progression by stabilizing POU2F1 through modulating its ubiquitination.

(A) qRT-PCR analysis was performed to confirm the transfection efficiency of POU2F1 rescue plasmids in CRC cells with altered USP18 expression.

(B, C) CCK-8 assay and colony formation assay were performed to evaluate the effects

of USP18 overexpression or knockdown on CRC cell proliferation and clonogenicity.
(D, E) CCK-8 assay and colony formation assay were performed to assess the effects of POU2F1 rescue in HCT116 and SW620 cells with altered USP18 expression.
(F) qRT-PCR analysis was performed to assess the effects of altered USP18 expression on POU2F1 mRNA levels.
(G) CHX chase assay was performed to evaluate the effects of USP18 on POU2F1 protein stability.
(H) Western blot analysis of POU2F1 ubiquitination was performed to assess the effects of USP18 on POU2F1 ubiquitination levels.
Data are presented as the mean ± SD of three independent experiments. ns, not significant; $^{**}P < 0.01$; $^{***}P < 0.001$; $^{****}P < 0.0001$.

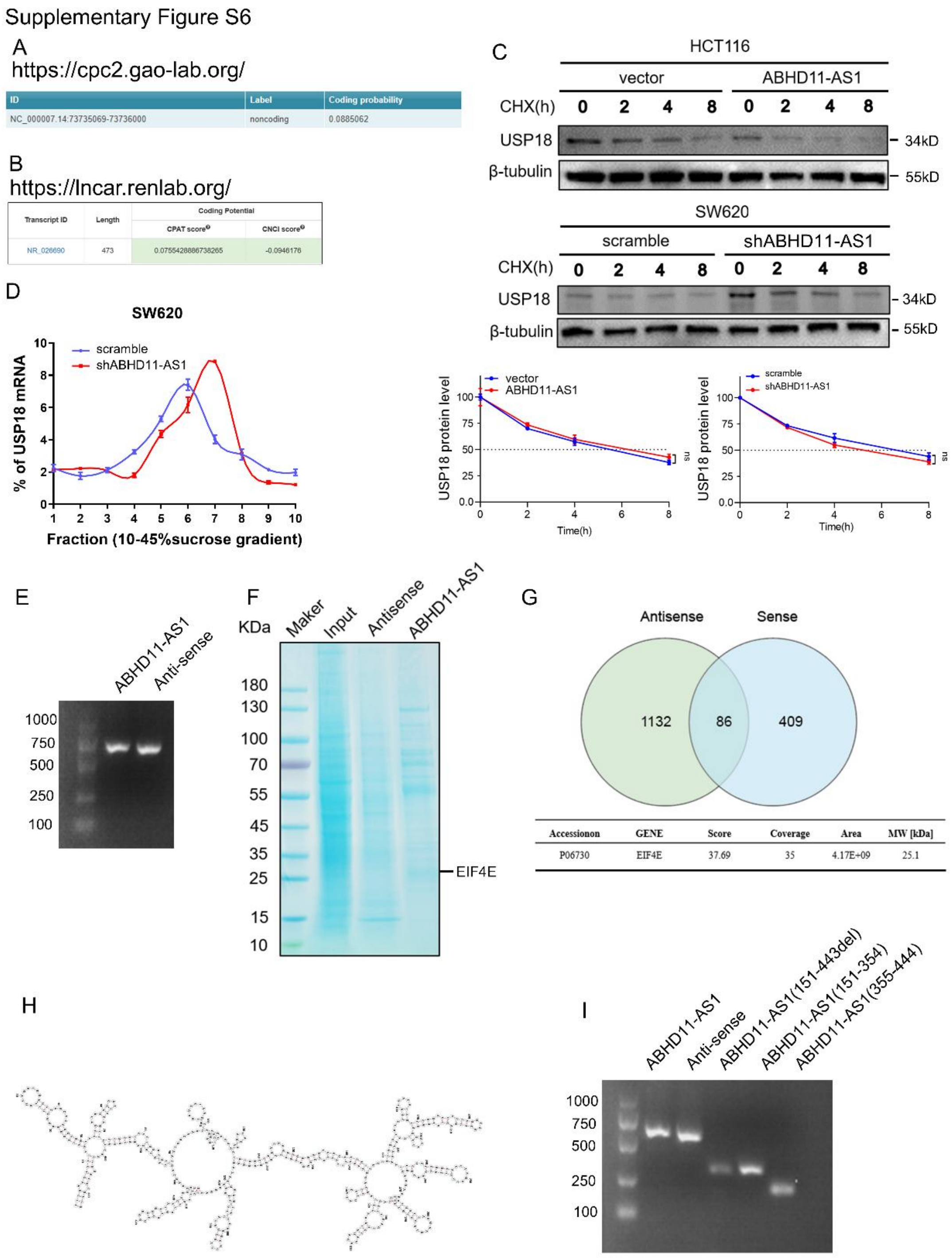

Supplementary Figure S6. ABHD11-AS1 does not affect USP18 protein stability while interacts with EIF4E

(A, B) Predicted coding potential of ABHD11-AS1 using two online tools: Coding Potential Calculator (CPC) (http://cpc.cbi.pku.edu.cn/) and lnCAR, a comprehensive resource for lncRNAs from Cancer Arrays (https://lncar.renlab.org/).

(C) CHX chase assays were performed to assess the effects of ABHD11-AS1 on USP18 protein stability.

(D) Sucrose gradient polysome fractionation analysis of the distribution of USP18 mRNA.

(E) DNA gel electrophoresis was performed to verify the in vitro transcription of ABHD11-AS1 and anti-sense RNA.
(F) RNA pulldown followed by SDS-PAGE and Coomassie Brilliant Blue staining was conducted to visualize ABHD11-AS1-associated proteins.
(G) Venn diagram summarizing the candidate ABHD11-AS1-interacting proteins identified by mass spectrometry analysis of pulldown fractions
(H) Secondary structure prediction of ABHD11-AS1 was performed using the ViennaRNA Web Services.
(I) DNA gel electrophoresis was performed to verify the in vitro transcription of full-length and truncated ABHD11-AS1 RNAs.
Data are presented as the mean ± SD of three independent experiments. ns, not significant.

Supplementary Figure S7

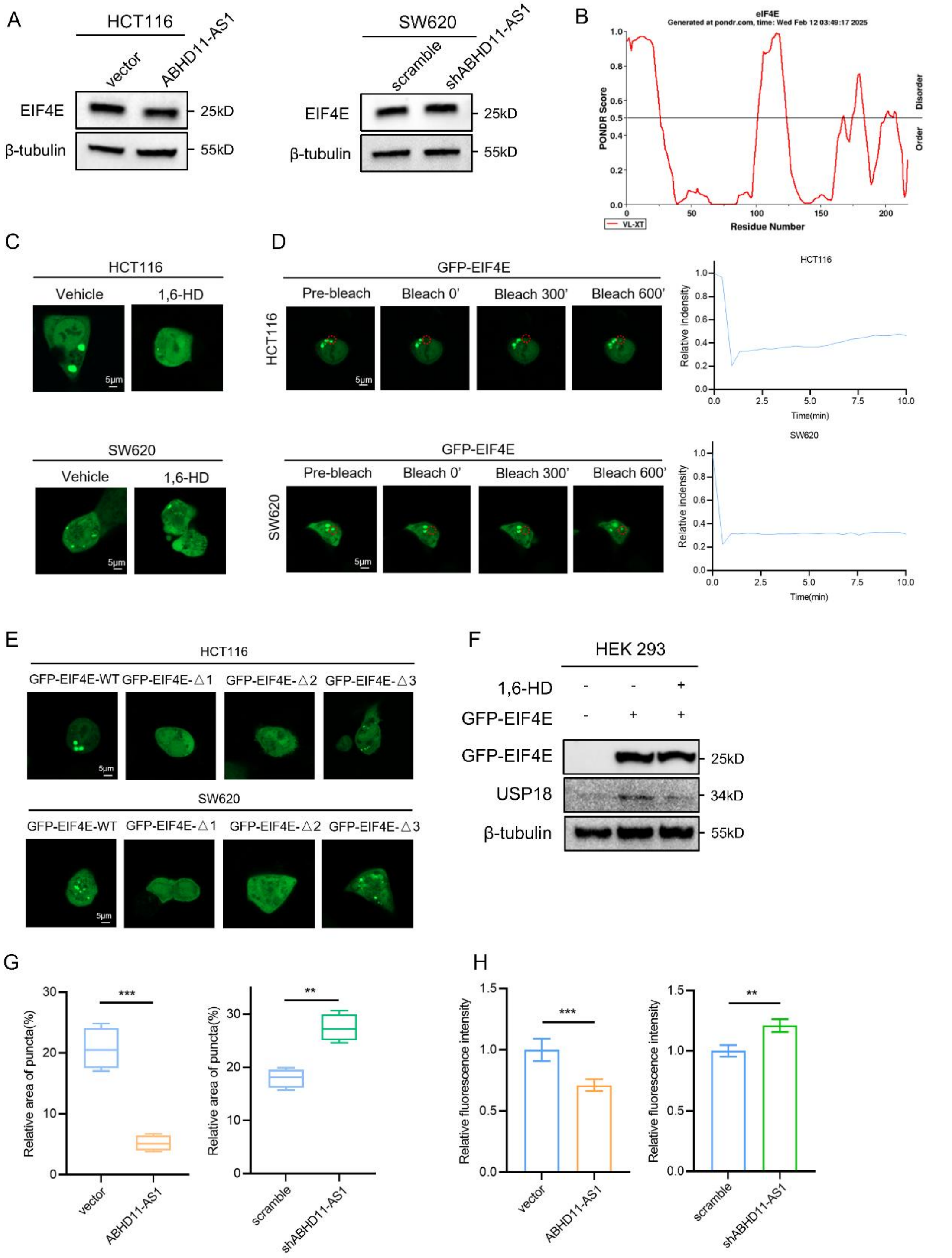

Supplementary Figure S7. EIF4E contains an intrinsically disordered region and its phase separation modulates USP18 protein expression.

(A) Western blot analysis was performed to assess the effects of ABHD11-AS1 overexpression or knockdown on total EIF4E protein levels in CRC cells.

(B) Intrinsic disorder propensity of EIF4E was analyzed using the PONDR.

(C) Representative fluorescence images of GFP–EIF4E condensates in CRC cell lines (HCT116 and SW620).

(D) FRAP analysis of GFP–EIF4E punctate condensates in HCT116 and SW620 cells.
(E) Fluorescence imaging of GFP–EIF4E wild-type (WT) and deletion mutants (Δ1, Δ2, Δ3) expressed in HCT116 and SW620 cells.
(F) HEK293 cells were treated with 0.5% 1,6-HD, and USP18 protein levels were assessed by western blotting.
(G–H) Quantification of EIF4E puncta size and fluorescence intensity after ABHD11-AS1 overexpression or knockdown, as shown in Figure 6E and Figure 6F.

Supplementary Figure S8

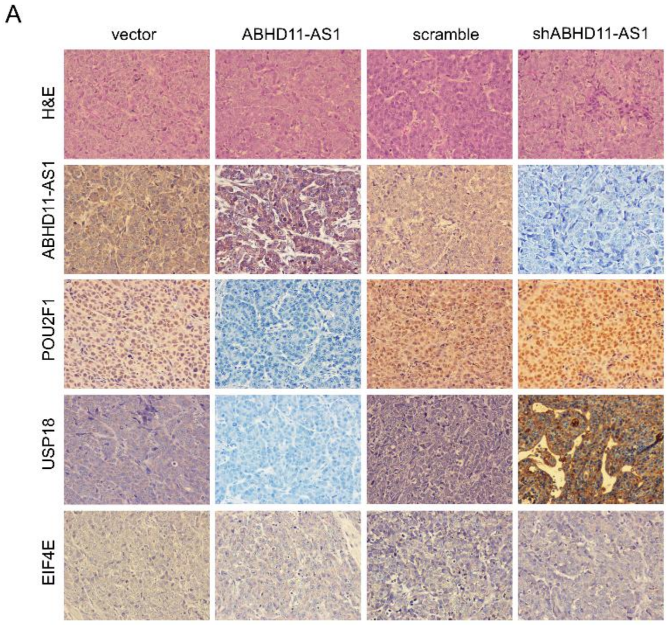

Supplementary Figure S8. Representative histological and immunohistochemical staining of xenograft tumors from the indicated groups.

(A)H&E staining shows tumor histomorphology. ISH/IHC staining was performed to detect ABHD11-AS1, POU2F1, USP18, and EIF4E expression in tumor sections.

Supplementary Table 1. The seqencence of shRNA used in this study.

Gene	NO.	Sequnence
	CON036	TTCTCCGAACGTGTCACGT
ABHD11-AS1	ABHD11-AS1-RNAi-1	GGGATGAAGCCATTGCCAAGA
	ABHD11-AS1-RNAi-2	ACCTCTTCCAGACAAGACTTG
	ABHD11-AS1-RNAi-3	GAGATGCAAAGCCAGGCTACA
	USP18-RNAi-1	GCCCTTGTTTGTCCAACATGA
USP18	USP18-RNAi-2	GCGATTCTCCATCAGGAATTC
	USP18-RNAi-3	GGACTCCTTGATTTGCGTTGA

Supplemental Table 2. Primers used in this study.

Primer names	sequence (5'-3')
ABHD11-AS1-F	GGATGAAGCCATTGCCAAGAAG
ABHD11-AS1-R	CCGCCTCAGCCTCAGTTT
USP18-F	ATTTGCGTTGACTGTGCCAT
USP18-R	CGCATGAGGTGGATTGTCAG
POU2F1-F	AAGCCAAGCCAACCTCCTAC
POU2F1-R	GGCTCAAGCCCTCAATTCCT
U6-F	GGAACGATACAGAGAAGATTAGC
U6-R	TGGAACGCTTCACGAATTTGCG
β-ACTB-F	CCTGGCACCCAGCACAAT
β-ACTB-R	GGGCCGGACTCGTCATAC
ABHD11-AS1 ISH probe	Dig-AAGUCUUGUCUGGAAGAGGUGUCUCACUC
ABHD11-AS1-sense-F	TAATACGACTCACTATAGGGCTAGC
ABHD11-AS1-sense-R	CCCCGAGTACCCTTGGC
ABHD11-AS1-Antisense-F	TAATACGACTCACTATAGGGCTAGC
ABHD11-AS1-Antisense-R	CCCTAAGTCCCAGCCCTTGA
ABHD11-AS1-del151-443-F	TAATACGACTCACTATAGGGCTAGC
ABHD11-AS1-del151-443-R	CCCCGAGTACCCTTGGC
ABHD11-AS1-151-354-F	TAATACGACTCACTATAGGGCTAGC
ABHD11-AS1-151-354-R	TAACTCCAGCAGCTGGGC
ABHD11-AS1-355-444-F	TAATACGACTCACTATAGGGCTAGC
ABHD11-AS1-355-444-R	CTGATTCTGGACCTGCTGC

F: Forword R: Reverse

Supplemental Table 3. Antibodies used in this study.

Antibodies A	Application, dilution	Catalogue No.	Supplier
POU2F1	WB,1:1000	ab178869	Abcam
POU2F1	WB,1:1000	10387-1-AP	Proteintech
USP18	WB,1:1000; IHC,1:200	ab161390	Abcam
USP18	WB,1:1000;	PA5-110555	Invitrogen
EIF4E	WB,1:1000	A19044	Abclonal
P-EIF4E	WB,1:1000	AP1024	Abclonal
ERK1/2	WB,1:1000	A4782	ABclonal
P-ERK1/2	WB,1:1000	AP0974	ABclonal
USP11	WB,1:1000	A19562	ABclonal
β-tubulin	WB,1:1000	AC106	Beyotime
Ub	WB,1:1000	AF1705	Beyotime
Flag	WB,1:1000	AE005	ABclonal
Myc	WB,1:1000	ab32	Abcam
GFP	WB,1:1000	AE078	ABclonal

Supplemental Table4. Differentially expressed proteins identified by proteomic profiling in HCT116 cells Transfected with ABHD11-AS1

Accession	Gene	log2(AB/Ve Ratio)	AB/Ve P value
A3KMH1	VWA8	3.427177	2.91E-05
O00219	HAS3	2.872454	0.000648
O14495	PLPP3	2.084949	0.000326
O14763	TNFRSF10B	1.761115	0.000536
O15031	PLXNB2	1.397365	0.000189
O15254	ACOX3	1.385818	0.004795
O15294	OGT	1.345056	0.000396
O15479	MAGEB2	1.302816	4.49E-05
O43157	PLXNB1	1.282321	0.003568
O43597	SPRY2	1.23634	0.00042
O60437	PPL	1.204579	0.003143
O60609	GFRA3	1.179702	0.030693
O75427	LRCH4	1.165944	0.006837
O75570	MTRF1	1.140582	4.09E-06
O75911	DHRS3	1.12307	8.98E-05
O95479	H6PD	1.12148	0.000232
O95810	CAVIN2	1.099026	0.000327
P00352	ALDH1A1	1.066813	0.000123
P00973	OAS1	1.063917	0.013887
P04181	OAT	0.58477	0.000532

P07093	SERPINE2	1.03956	0.000799
P08034	GJB1	1.027649	0.010734
P08133	ANXA6	1.027649	0.001835
P08138	NGFR	1.025879	9.41E-05
P08473	MME	1.017851	0.000659
P10586	PTPRF	0.918997	0.001913
P11166	SLC2A1	0.91831	0.001621
P11169	SLC2A3	0.91663	0.000811
P15328	FOLR1	0.912343	0.00162
P16219	ACADS	0.897395	0.000635
P16444	DPEP1	0.882995	0.000365
P17028	ZNF24	0.879784	0.000381
P17301	ITGA2	0.872317	0.016799
P27338	MAOB	0.83996	0.019274
P32004	L1CAM	0.834469	0.000569
P33908	MAN1A1	0.826925	0.035258
P37173	TGFBR2	0.823179	0.000745
P43121	MCAM	0.82122	0.009547
P46939	UTRN	0.807685	0.003646
P48200	IREB2	0.806778	0.003917
P49006	MARCKSL1	0.803392	0.00301
P50225	SULT1A1	0.80331	1.10E-06
P50895	BCAM	0.802317	0.00962
P53794	SLC5A3	0.799419	0.005455
P54277	PMS1	0.793188	0.004444
P54753	EPHB3	0.787683	0.017105
P55290	CDH13	0.773068	0.001039
P57723	PCBP4	0.772055	8.04E-05
P58107	EPPK1	0.767909	0.00094
P98172	EFNB1	0.765195	9.55E-07
Q01628	IFITM3	0.764601	0.002247
Q08431	MFGE8	0.759667	0.003275
Q12955	ANK3	0.750949	0.000962
Q13642	FHL1	0.733615	0.001473
Q14194	CRMP1	0.715278	0.000525
Q14451	GRB7	0.715191	0.001011
Q14592	ZNF460	0.707171	0.012962
Q15011	HERPUD1	0.707083	0.00735
Q15274	QPRT	0.69813	0.000334
Q15434	RBMS2	0.689478	0.001346
Q3YBM2	TMEM176B	0.687777	0.018875
Q4L180	FILIP1L	0.684729	0.002333
Q53RT3	ASPRV1	0.681674	0.039222
Q5SRI9	MANEA	0.675545	0.008461

Q5W0Z9	ZDHHC20	0.67428	0.000282
Q658P3	STEAP3	0.673647	0.009187
Q6NSJ2	PHLDB3	0.671746	0.003435
Q6PCE3	PGM2L1	0.67084	0.004043
Q70CQ2	USP34	0.829931	0.004816
Q7Z3F1	GPR155	0.666847	0.011573
Q7Z699	SPRED1	0.666211	0.000642
Q86VI3	IQGAP3	0.656451	0.001186
Q86WV6	STING1	0.655352	0.000176
Q86YS6	RAB43	0.645241	0.029974
Q8IUS5	EPHX4	0.641084	4.09E-05
Q8IXQ5	KLHL7	0.637378	0.009634
Q8IZW8	TNS4	0.634036	0.01995
Q8N0X4	CLYBL	0.631337	0.016845
Q8N3D4	EHBP1L1	0.626205	0.009307
Q8N4Q0	ZADH2	0.624616	0.000406
Q8N4S9	MARVELD2	0.622462	0.000648
Q8N4T8	CBR4	0.618051	0.001432
Q8N680	ZBTB2	0.61711	0.0049
Q8N726	CDKN2A	0.615416	0.000522
Q8TAA5	GRPEL2	0.595313	0.005199
Q8WVX9	FAR1	0.592923	0.030976
Q8WXH0	SYNE2	0.591296	0.000228
Q92485	SMPDL3B	0.587269	0.01205
Q92558	WASF1	0.586212	0.00291
A9UHW6	MIF4GD	-0.88843	0.003611
O14965	AURKA	-1.30187	0.005605
O15460	P4HA2	-1.58381	0.000356
O43572	AKAP10	-0.78265	0.019553
O75496	GMNN	-1.51994	0.010515
O95817	BAG3	-1.55343	0.000133
P04792	HSPB1	-0.99712	0.000783
P0DMV9	HSPA1B	-1.32301	0.003863
P10412	H1-4	-1.47033	0.024813
P14859	POU2F1	-1.47915	0.00635
P35251	RFC1	-1.09573	0.023296
P39060	COL18A1	-1.14274	0.000122
P51692	STAT5B	-1.31007	0.000228
P51784	USP11	-1.31007	0.000228
P53999	SUB1	-0.68084	0.040153
Q00653	NFKB2	-1.2643	0.000394
Q12834	CDC20	-1.70887	0.000241
Q13015	MLLT11	-1.21257	0.006414
Q15036	SNX17	-1.31581	0.000472

Q16644	MAPKAPK3	-0.59575	0.0187
Q2TB90	HKDC1	-2.16424	0.001139
Q53EU6	GPAT3	-1.2923	0.00383
Q5NDL2	EOGT	-1.08039	0.009801
Q66PJ3	ARL6IP4	-0.89593	0.03005
Q676U5	ATG16L1	-0.76195	0.026562
Q7RTP6	MICAL3	-0.83882	0.008106
Q7Z2K8	GPRIN1	-0.69222	0.022648
Q8IV38	ANKMY2	-0.96325	0.000357
Q8IVD9	NUDCD3	-1.04275	2.16E-05
Q8IXU6	SLC35F2	-0.74371	0.042485
Q8IZT6	ASPM	-1.39327	0.024004
Q8N5F7	NKAP	-0.95568	0.018267
Q8N5M4	TTC9C	-0.74977	0.032315
Q8N9N8	EIF1AD	-1.06703	0.001456
Q8TDX7	NEK7	-0.96353	1.30E-05
Q96CN9	GCC1	-1.45087	0.017504
Q96ME7	ZNF512	-2.03919	0.005148
Q9BXS6	NUSAP1	-1.09111	0.005113
Q9BYN0	SRXN1	-1.1131	0.002939
Q9H0A8	COMMD4	-1.02974	0.000199
Q9H0W8	SMG9	-0.82674	0.007097
Q9H173	SIL1	-1.1594	0.020656
Q9H2G4	TSPYL2	-1.14114	0.005923
Q9H977	WDR54	-0.94148	0.036159
Q9HC98	NEK6	-2.46355	0.022288
Q9NX01	TXNL4B	-0.88737	0.009854
Q9NX31	OSER1	-0.87832	0.00078
Q9UBT7	CTNNAL1	-0.72309	0.033037
Q9UGK3	STAP2	-0.89916	0.002751
Q9UMW8	USP18	-1.2723	0.006277

Supplemental Table 5. Potential ABHD11-AS1 interacting proteins in HCT116 cells using the LC-MS/MS method after pulldown by the biotin-labeled ABHD11-AS1 probe.

Accession	Gene	Sum PEP Score	MW [kDa]	Abundances (Normalized)
Q9NYF8	BCLAF1	108.113	106.1	3E+09
Q92804	TAF15	95.136	61.8	1.74E+09
Q9UN86	G3BP2	85.747	54.1	4.77E+09
P68363	TUBA1B	80.58	50.1	1.28E+09
Q52LJ0	FAM98B	70.789	45.5	3.27E+08
Q14444	CAPRIN1	68.039	78.3	2.85E+09
Q9Y224	RTRAF	50.456	28.1	9.71E+08
Q9BY77	POLDIP3	47.859	46.1	7.76E+08
Q8WWM7	ATXN2L	47.838	113.3	5.16E+08
Q86V81	ALYREF	44.786	26.9	2.73E+09
Q10570	CPSF1	42.924	160.8	1.78E+08
O14979	HNRNPDL	35.767	46.4	3.47E+08
P06730	EIF4E	35.377	25.1	4.17E+09
Q15424	SAFB	33.887	102.6	7.39E+08
P98179	RBM3	31.286	17.2	3.36E+08
P51116	FXR2	27.183	74.2	95078622
Q06787	FMR1	26.606	71.1	1.76E+08
Q13151	HNRNPA0	18.707	30.8	1.95E+08
Q9NUL7	DDX28	14.953	59.5	1.13E+08
Q13642	FHL1	14.174	36.2	1.74E+08
Q9Y3Y2	CHTOP	13.921	26.4	98017080
Q01804	OTUD4	13.326	124	77767116
O00425	IGF2BP3	13.192	63.7	8621770
Q86YZ3	HRNR	11.994	282.2	11602116
Q14011	CIRBP	11.678	18.6	32232278
O15131	KPNA5	11.594	60.6	52749906
P49916	LIG3	11.064	112.8	31973995
Q08945	SSRP1	10.339	81	36065061
Q14694	USP10	10.176	87.1	1.02E+08
Q13573	SNW1	9.411	61.5	21754982
P23588	EIF4B	9.383	69.1	24465670
P00918	CA2	9.361	29.2	19197602
P12277	CKB	8.328	42.6	2.05E+08
Q16527	CSRP2	7.82	20.9	1.92E+08
Q9P2N5	RBM27	7.699	118.6	3.37E+08
Q86U42	PABPN1	7.661	32.7	41678365
Q96EP5	DAZAP1	7.602	43.4	70030460
Q9UKB1	FBXW11	7.254	62.1	856731.5
O43148	RNMT	7.198	54.8	58746719

P01023	A2M	6.688	163.2	1.49E+08
Q9P2I0	CPSF2	6.328	88.4	37067781
Q9HC62	SENP2	6.034	67.8	11025893
Q8WXF1	PSPC1	6.006	58.7	18890326
Q6P5R6	RPL22L1	5.666	14.6	29413646
Q13410	BTN1A1	5.43	58.9	18499330
P78332	RBM6	5.159	128.6	37349922
P0C0L4	C4A	5.13	192.7	68142824
P69905	HBA1; HBA2	5.051	15.2	33356190
P27816	MAP4	4.721	120.9	19664640
A6NMY6	ANXA2P2	4.524	38.6	8923962
O14654	IRS4	4.277	133.7	43418371
Q99700	ATXN2	4.199	140.2	8968326
P06858	LPL	4.144	53.1	57502644
P08670	VIM	4.119	53.6	2.89E+08
Q9NX20	MRPL16	3.54	28.4	28422454
Q6DD87	ZNF787	3.532	40.4	14001240
P01024	C3	3.512	187	77662784
P20742	PZP	3.496	163.8	6.46E+09
P55854	SUMO3	3.486	11.6	5.11E+08
P56270	MAZ	3.471	48.6	11766515
Q9NNW5	WDR6	3.236	121.6	939426.5
Q9UK55	SERPINA10	2.92	50.7	11938458
P00450	CP	2.772	122.1	8119936
Q7L2E3	DHX30	2.727	133.9	7916557
P05413	FABP3	2.414	14.8	14926973
P15291	B4GALT1	2.357	43.9	15969182
P02749	APOH	2.307	38.3	20234336
Q7Z2W4	ZC3HAV1	2.307	101.4	24481536
P25490	YY1	2.287	44.7	11814873
Q4VCS5	AMOT	2.144	118	15387067
Q8N684	CPSF7	2.119	52	7765550
Q9NY12	GAR1	2.066	22.3	16648792
Q96MU7	YTHDC1	1.918	84.6	5742305
P01624	IGKV3-15	1.712	12.5	44520320
P16671	CD36	1.692	53	36563608
Q9UGN5	PARP2	1.657	66.2	10639618
Q9H2G4	TSPYL2	1.301	79.4	81478472
Q9GZX5	ZNF350	1.156	60	1.54E+10
Q05397	PTK2	1.131	119.2	38494324
Q7Z5H4	VN1R5	1.06	40.8	47034372
Q8N5Z0	AADAT	0.933	47.3	23248108
Q6PI26	SHQ1	0.89	65.1	7.66E+08
P02787	TF	0.889	77	1.34E+08

B9A064	IGLL5	0.866	23	1.84E+08
Q9NV31	IMP3	0.809	21.8	4.19E+08
